# Supplementary material for: Lifetime air pollution exposure from prenatal to 18 years and cardiovascular health in young adulthood: findings from a UK birth cohort
Source: Environ Int. Author manuscript; Available in PMC 2026 Jan 30. (PMC7618702; doi:10.1016/j.envint.2025.109754)
Supplement: Supplementary material [file EMS212150-supplement-Supplementary_material.pdf]

Lifetime air pollution exposure from prenatal to 18 years and cardiovascular health in young adulthood: findings from a UK birth cohort

Supplementary methods

Cardiovascular health markers assessment

Carotid intima-media thickness (CIMT) was measured using B-mode ultrasound from ZONARE z.oneUltra convertible ultrasound machines. Both left and right carotid arteries were imaged three times 1-2 cm proximal to the carotid bifurcation. Ten second cine-loops were recorded and analysed offline by trained practitioners. CIMT was recorded to the nearest 0.01 mm, and the average of the right and left side measures was used.

For brachial (peripheral) blood pressure and heart rate, individuals wore appropriate loose sleeves on the upper-arm and were asked to sit in a chair and rest each arm on a table. The circumference of one upper arm was measured in cm. The appropriate sized blood pressure cuff was fitted to the person's upper arm. Brachial blood pressure was measured after resting in a seated position for 5 minutes, using an Omron 705 IT semiautomatic oscillometric recorder (Omron Electronic Components Europe BV). Blood pressure and heart rate were measured three times in the right arm, and the average of the second and third readings was used.

Central blood pressure and central augmentation index (AIx) were measured using radial artery tonometry with a SphygmoCor Px Pulse Wave Analysis System (Atcor Medical). Recordings were calibrated using the brachial blood pressure (measured with the Omron 705 IT in the same session) according to manufacturer's instructions. Central blood pressure (mmHg) and central AIx (%) were measured twice and the average was calculated.

Carotid to femoral pulse wave velocity (PWV) was assessed using Vicorder portable physiologic vascular testing equipment. PWV velocity was calculated from measurements of pulse transit time and distance travelled by the pulse between carotid to femoral. For PWV, a cuff was placed over the right carotid artery in the participant's neck, while another was located over the femoral artery in their upper right thigh. The distance between the participant's suprasternal notch and the top of the thigh cuff was measured, as was the distance between their suprasternal notch and the bottom of the neck cuff on the right side. PWV and transit time were automatically computed. PWV was measured three times and recorded in meters per second (m/s); the average of these measures was used.

Covariates

Maternal age, education and ethnicity were assessed at recruitment. Maternal age at delivery was calculated from the mother's date of birth (obtained at enrolment) and the date of delivery. Maternal level of education was based on the highest ongoing or completed education. It was classified as low, medium and high according to the International Standard Classification of Education 97/2011 (ISCED-97/2011). High education corresponded to short cycle tertiary, Bachelor, Masters, Doctoral or equivalent (ISCED2011: 5-8, ISCED-97: 5-6); medium education corresponded to upper secondary, or post-secondary non-tertiary (ISCED-2011: 3-4, ISCED-97: 3-4); and low education corresponded to no education, early childhood, pre-primary, primary, lower secondary or second stage of basic education (ISCED-2011: 0-2, ISCED-97: 0-2). Maternal ethnicity was self-reported at enrolment and recoded into White and non-White.

Index of multiple deprivation (IMD), a measure of relative deprivation, was based on data from the Ministry of Housing, Communities & Local Government (UK Government) from 2015 and linked to geocoded addresses at recruitment. It combined weighted information from seven domains to produce an overall relative measure of deprivation. The domains and their weights were the following: income deprivation (22.5%), employment deprivation (22.5%), education, skills and training deprivation (13.5%), health deprivation and disability (13.5%), crime (9.3%), barriers to housing and services (9.3%), and living environment deprivation (9.3%). IMD was categorised in quintiles, with the first corresponding to the least deprived and the last to the most deprived.

Sex was obtained from the birth notification at enrolment. Age at outcome assessment (complete years) was calculated from date of birth and date of attendance at the research clinic.

Supplementary Tables and Figures

Supplementary Table 1. Descriptive statistics of air pollution from pregnancy to 18 years.

Supplementary Table 2. Association between socioeconomic indicators and air pollutants averages from pregnancy to 18 years.

Supplementary Table 3. Model fit parameters for group-based trajectory models of air pollution from pregnancy to 18 years.

Supplementary Table 4. Mean levels of fine particulate matter ($PM_{2.5}$) (μg/m^3) in each age by latent classes.

Supplementary Table 5. Mean levels of nitrogen dioxide (NO_2) (μg/m^3) in each age by latent classes.

Supplementary Table 6. Mean levels of black carbon (10^{-5}/m) in each age by latent classes.

Supplementary Table 7. Fine particulate matter ($PM_{2.5}$) classes distribution by socioeconomic indicators.

Supplementary Table 8. Nitrogen dioxide (NO_2) classes distribution by socioeconomic indicators.

Supplementary Table 9. Black carbon (BC) classes distribution by socioeconomic indicators.

Supplementary Table 10. Association between fine particulate matter ($PM_{2.5}$) from pregnancy to 18 years with cardiovascular health indicators at age 18 years.

Supplementary Table 11. Association between nitrogen dioxide (NO_2) from pregnancy to 18 years with cardiovascular health indicators at age 18 years.

Supplementary Table 12. Association between black carbon (BC) from pregnancy to 18 years with cardiovascular health indicators at age 18 years.

Supplementary Table 13. Association between air pollution at different age ranges and cardiovascular health markers at age 18 years.

Supplementary Table 14. Association between fine particulate matter ($PM_{2.5}$) and cardiovascular health markers at 18 years in males and females.

Supplementary Table 15. Association between nitrogen dioxide (NO_2) and cardiovascular health markers at 18 years in males and females.

Supplementary Table 16. Association between black carbon (BC) and cardiovascular health markers at 18 years in males and females.

Supplementary Table 17. Association between fine particulate matter ($PM_{2.5}$) and cardiovascular health markers at 18 years in non-movers and movers.

Supplementary Table 18. Association between nitrogen dioxide (NO_2) and cardiovascular health markers at 18 years in non-movers and movers.

Supplementary Table 19. Association between black carbon (BC) and cardiovascular health markers at 18 years in non-movers and movers.

Supplementary Table 20. Association between air pollutants from pregnancy to 18 years with birthweight and maternal pre-pregnancy body mass index (BMI).

Supplementary Figure 1. Flow chart of participants.

Supplementary Figure 2. Assumed causal diagram of the association between air pollution and cardiovascular health markers.

Supplementary Figure 3. Correlation between air pollutants across ages from pregnancy (age 0) to 18 years.

Supplementary Figure 4. Correlation between air pollutants across age ranges.

Supplementary Figure 5. Association between air pollution at different age ranges and cardiovascular health markers at age 18 years.

Supplementary Table 1. Descriptive statistics of air pollution from pregnancy to 18 years.

Pollutant/ age	N	Mean	SD	Median	p25	p75	IQR
$PM_{2.5}$ (μg/m^3)							
0	3,762	29.75	2.53	29.26	28.10	31.81	3.71
1	3,760	28.89	1.83	29.07	28.03	30.03	2.00
2	3,760	25.94	2.99	25.03	23.88	28.63	4.75
3	3,758	24.04	1.58	24.25	23.26	24.91	1.65
4	3,754	26.14	2.41	26.40	24.42	27.91	3.49
5	3,754	26.15	2.11	26.25	25.20	27.54	2.34
6	3,754	21.75	3.24	20.52	19.57	25.02	5.45
7	3,751	19.22	1.46	19.49	18.57	20.15	1.58
8	3,755	18.24	1.49	18.40	17.54	19.23	1.69
9	3,759	19.01	1.74	19.18	18.03	20.28	2.25
10	3,755	19.32	1.54	19.51	18.71	20.28	1.57
11	3,755	21.30	2.41	21.74	19.51	23.31	3.80
12	3,756	19.69	2.67	18.90	17.99	22.21	4.22
13	3,758	17.88	1.43	18.24	17.26	18.75	1.49
14	3,760	18.32	1.54	18.62	17.64	19.27	1.63
15	3,756	18.26	1.51	18.57	17.58	19.20	1.62
16	3,755	16.08	1.98	15.69	14.85	17.68	2.83
17	3,753	14.08	1.31	14.12	13.39	15.01	1.62
18	3,754	14.04	1.20	14.20	13.49	14.80	1.31
Average	3,767	20.95	1.38	21.11	20.26	21.89	1.63
Average[#]	3,717	20.96	1.37	21.12	20.27	21.89	1.63
NO_2 (μg/m^3)							
0	3,766	58.44	9.07	59.36	52.99	64.45	11.45
1	3,760	55.86	8.78	56.85	50.68	61.71	11.03
2	3,760	49.69	8.82	50.05	44.15	55.54	11.39
3	3,754	47.10	7.65	48.30	42.72	52.28	9.56
4	3,754	50.74	8.84	51.61	45.58	56.91	11.32
5	3,751	50.65	8.71	51.81	45.77	56.65	10.88
6	3,752	42.61	8.97	42.29	36.83	48.53	11.70
7	3,750	38.22	6.62	39.25	34.44	42.96	8.52
8	3,754	35.31	6.53	36.15	31.37	39.73	8.35
9	3,757	37.81	7.51	38.41	32.93	43.40	10.47
10	3,754	36.66	7.04	37.28	32.28	41.40	9.12
11	3,754	36.29	6.70	37.18	32.28	41.04	8.76
12	3,756	33.83	6.58	34.34	29.76	38.25	8.48
13	3,758	31.60	5.76	32.40	28.30	35.81	7.51
14	3,760	30.99	5.68	31.80	27.71	35.11	7.40
15	3,756	30.71	5.64	31.55	27.46	34.85	7.39
16	3,755	29.65	5.54	30.47	26.28	33.61	7.33
17	3,753	26.58	5.27	27.12	23.23	30.15	6.92
18	3,754	26.82	5.18	27.40	23.60	30.46	6.86
Average	3,767	39.45	6.02	40.15	35.78	43.74	7.96
Average[#]	3,715	39.45	6.00	40.15	35.79	43.74	7.95

BC (10^{-5}/m)							
0	3,766	1.77	0.39	1.80	1.45	2.06	0.61
1	3,760	1.78	0.38	1.80	1.48	2.10	0.62
2	3,760	1.62	0.38	1.62	1.31	1.88	0.57
3	3,758	1.52	0.33	1.54	1.26	1.81	0.55
4	3,755	1.76	0.43	1.76	1.44	2.08	0.64
5	3,754	1.85	0.42	1.87	1.51	2.21	0.70
6	3,754	1.56	0.41	1.54	1.23	1.82	0.58
7	3,751	1.37	0.31	1.37	1.11	1.64	0.53
8	3,755	1.44	0.34	1.44	1.17	1.71	0.55
9	3,759	1.71	0.43	1.69	1.38	2.02	0.65
10	3,755	1.71	0.41	1.69	1.38	2.03	0.65
11	3,755	1.82	0.45	1.79	1.46	2.14	0.68
12	3,756	1.63	0.44	1.60	1.28	1.91	0.63
13	3,758	1.49	0.35	1.49	1.21	1.80	0.59
14	3,760	1.55	0.38	1.54	1.25	1.86	0.61
15	3,756	1.46	0.35	1.44	1.18	1.74	0.57
16	3,755	1.34	0.32	1.33	1.08	1.61	0.53
17	3,753	1.24	0.30	1.23	0.99	1.49	0.50
18	3,754	1.36	0.34	1.34	1.08	1.61	0.53
Average	3,767	1.58	0.33	1.57	1.31	1.86	0.55
Average[#]	3,722	1.58	0.33	1.57	1.31	1.87	0.55

[#] complete cases

BC: black carbon; IQR: interquartile range; NO_2: nitrogen dioxide; p25: 25th percentile, p75: 75th percentile, $PM_{2.5}$: fine particulate matter, SD: standard deviation

Supplementary Table 2. Association between socioeconomic indicators and air pollutants averages from pregnancy to 18 years.

Socioeconomic indicators	Difference in mean (95% CI)		
	$PM_{2.5}$	NO_2	BC
Maternal education	p<0.001	p<0.001	p=0.001
Low	Reference	Reference	Reference
Medium	-0.28 (-0.42, -0.14)	-1.09 (-1.70, -0.47)	-0.05 (-0.09, -0.02)
High	-0.67 (-0.84, -0.51)	-0.98 (-1.70, -0.26)	-0.05 (-0.09, -0.01)
Area deprivation	p<0.001	p<0.001	p<0.001
1st Q (least deprived)	Reference	Reference	Reference
2nd quintile	-0.19 (-0.33, -0.05)	-1.00 (-1.59, -0.42)	0.01 (-0.02, 0.04)
3rd quintile	0.10 (-0.03, 0.23)	1.37 (0.81, 1.93)	0.14 (0.11, 0.17)
4th quintile	0.42 (0.28, 0.56)	3.46 (2.87, 4.05)	0.27 (0.23, 0.30)
5th Q (most deprived)	0.54 (0.40, 0.68)	3.03 (2.43, 3.62)	0.26 (0.23, 0.29)
Moving status from pregnancy to 18 years	p<0.001	p<0.001	p<0.001
Moved address	-1.55 (-1.66, -1.44)	-4.82 (-5.32, -4.32)	-0.26 (-0.29, -0.23)

BC: black carbon; NO_2: nitrogen dioxide; $PM_{2.5}$: fine particulate matter; Q: quintile

Supplementary Table 3. Model fit parameters for group-based trajectory models of air pollution from pregnancy to 18 years.

Air pollutant	Classes	BIC	AIC	APP	Entropy	Class 1 (%)	Class 2 (%)	Class 3 (%)	Class 4 (%)	Class 5 (%)	Class 6 (%)
$PM_{2.5}$	1	337164.5	337139.6	NA	1.000	100.00	NA	NA	NA	NA	NA
	2	327482.0	327432.2	0.960	0.894	23.97	76.03	NA	NA	NA	NA
	3	324651.2	324576.4	0.922	0.817	11.87	33.98	54.15	NA	NA	NA
	**4**	**323854.4**	**323754.7**	**0.908**	**0.846**	**7.51**	**34.11**	**53.04**	**5.34**	**NA**	**NA**
	5	323145.9	323021.2	0.879	0.807	5.23	52.24	4.91	20.92	16.70	NA
	6	322959.6	322809.9	0.859	0.781	3.34	27.48	12.95	46.08	4.99	5.15
NO_2	1	490896.5	490871.5	NA	1.000	100.00	NA	NA	NA	NA	NA
	2	456193.6	456143.7	0.987	0.956	60.76	39.24	NA	NA	NA	NA
	3	443395.4	443320.6	0.976	0.946	41.17	42.74	16.09	NA	NA	NA
	4	437908.9	437809.2	0.961	0.929	29.57	12.82	20.28	37.32	NA	NA
	5	432431.3	432306.6	0.963	0.941	11.55	25.22	36.24	19.88	7.11	NA
	**6**	**429934.1**	**429784.5**	**0.951**	**0.926**	**30.00**	**26.86**	**9.69**	**17.23**	**9.45**	**6.77**
BC	1	72506.27	72481.33	NA	1.000	100.00	NA	NA	NA	NA	NA
	2	30689.42	30639.55	0.989	0.963	51.69	48.31	NA	NA	NA	NA
	3	19442.2	19367.39	0.974	0.946	33.71	34.54	31.75	NA	NA	NA
	4	15313.33	15213.59	0.960	0.931	26.92	27.32	28.01	17.76	NA	NA
	5	11434.29	11309.61	0.959	0.940	7.43	24.63	23.17	27.48	17.28	NA
	**6**	**9603.894**	**9454.277**	**0.940**	**0.917**	**22.25**	**21.05**	**21.08**	**13.49**	**14.65**	**7.49**

AIC: Akaike Information Criterion; APP: average posterior probability; BC: black carbon; BIC: Bayesian Information Criterion; NO_2: nitrogen dioxide; $PM_{2.5}$: fine particulate matter

Supplementary Table 4. Mean levels of fine particulate matter (PM2.5) (µg/m3) in each age by latent classes.

Age	Low, decreasing (6.6%)	Average-low, decreasing (18.0%)	Average-high, decreasing (53.5%)	High, decreasing (21.9%)
			Mean (SD) µg/m^3	
0	27.41 (2.99)	28.04 (2.21)	29.48 (1.78)	32.50 (1.79)
1	26.51 (2.90)	27.72 (1.71)	29.13 (1.33)	29.98 (1.41)
2	22.47 (2.84)	24.07 (2.38)	25.67 (2.19)	29.19 (2.22)
3	20.97 (2.09)	22.90 (1.31)	24.25 (0.92)	25.36 (0.95)
4	22.63 (2.35)	25.26 (2.09)	26.73 (2.08)	26.47 (2.32)
5	21.55 (1.68)	24.47 (1.44)	26.32 (1.05)	28.47 (0.94)
6	17.31 (2.02)	19.70 (2.10)	21.55 (2.44)	25.27 (2.56)
7	15.82 (0.89)	17.96 (0.98)	19.47 (0.65)	20.70 (0.69)
8	14.94 (0.94)	16.93 (0.95)	18.42 (0.70)	19.85 (0.71)
9	15.88 (1.18)	18.03 (1.54)	19.49 (1.37)	19.58 (1.48)
10	15.92 (1.07)	17.94 (1.16)	19.57 (0.76)	20.85 (0.67)
11	17.92 (1.72)	20.27 (2.23)	21.96 (2.13)	21.52 (2.25)
12	15.70 (1.55)	17.69 (1.57)	19.60 (1.88)	22.73 (1.99)
13	14.77 (0.80)	16.59 (1.07)	18.15 (0.75)	19.22 (0.72)
14	15.27 (1.00)	17.12 (1.32)	18.70 (1.04)	19.31 (1.06)
15	15.03 (0.89)	16.86 (1.09)	18.51 (0.80)	19.76 (0.74)
16	12.98 (1.20)	14.50 (1.21)	16.05 (1.33)	18.34 (1.37)
17	11.58 (0.82)	12.90 (0.83)	14.18 (0.73)	15.55 (0.75)
18	11.83 (0.93)	13.15 (1.04)	14.32 (0.87)	14.75 (0.89)
Overall	17.71 (0.68)	19.60 (0.50)	21.13 (0.49)	22.60 (0.54)

SD: standard deviation

Supplementary Table 5. Mean levels of nitrogen dioxide (NO_2) (μg/m^3) in each age by latent classes.

Age	Low, decreasing (9.7%)	Average-low, decreasing (17.2%)	Average, decreasing (26.9%)	Average-high, decreasing (30.0%)	High, decreasing (9.4%)	High, decreasing fast (6.8%)
	Mean (SD) μg/m^3					
0	44.10 (8.75)	50.35 (5.27)	57.40 (4.38)	63.78 (4.21)	70.00 (4.60)	63.88 (6.54)
1	40.87 (7.54)	47.94 (4.51)	55.01 (3.84)	61.21 (3.89)	67.24 (4.15)	61.16 (6.07)
2	35.05 (5.90)	42.22 (4.13)	48.73 (4.22)	54.88 (4.76)	61.70 (5.16)	53.59 (7.69)
3	33.00 (4.82)	40.88 (3.84)	46.83 (3.29)	51.90 (3.32)	57.08 (3.79)	48.91 (7.58)
4	35.26 (5.15)	44.56 (4.73)	50.91 (4.74)	55.97 (5.00)	61.11 (5.87)	50.32 (10.10)
5	34.66 (4.19)	43.97 (3.71)	50.70 (3.09)	56.49 (3.10)	63.02 (3.44)	47.25 (10.51)
6	28.90 (4.86)	36.46 (4.49)	42.38 (4.85)	48.02 (5.69)	54.77 (6.27)	37.88 (9.46)
7	26.43 (3.27)	33.64 (2.71)	38.57 (2.39)	42.83 (2.36)	47.28 (3.08)	32.20 (7.47)
8	24.32 (3.17)	31.11 (2.81)	35.58 (2.54)	39.76 (2.84)	44.71 (3.11)	27.71 (5.86)
9	26.41 (3.82)	34.35 (4.33)	38.73 (4.68)	42.15 (5.06)	45.86 (5.80)	28.70 (6.48)
10	25.20 (3.36)	32.51 (3.17)	36.87 (2.87)	41.32 (3.43)	47.06 (3.40)	27.41 (5.08)
11	25.34 (3.26)	33.07 (3.42)	36.91 (3.27)	40.60 (3.44)	44.90 (3.86)	26.36 (4.37)
12	23.38 (3.27)	30.31 (3.18)	34.03 (2.91)	38.19 (3.37)	43.24 (3.58)	24.35 (3.67)
13	22.02 (2.70)	28.80 (2.87)	32.01 (2.36)	35.47 (2.57)	39.44 (3.08)	22.67 (3.22)
14	21.67 (2.72)	28.19 (2.83)	31.38 (2.46)	34.77 (2.69)	38.76 (2.93)	22.27 (3.16)
15	21.55 (2.63)	27.95 (2.87)	31.08 (2.63)	34.45 (2.74)	38.26 (3.15)	22.13 (3.17)
16	20.82 (2.66)	26.78 (2.85)	29.93 (2.66)	33.32 (2.72)	37.24 (3.03)	21.45 (3.11)
17	18.60 (2.63)	23.86 (2.86)	26.69 (2.81)	29.94 (2.92)	33.88 (3.29)	19.34 (3.10)
18	19.07 (2.79)	24.57 (3.18)	27.28 (3.01)	29.93 (3.28)	32.81 (3.72)	19.52 (3.45)
Overall	27.76 (2.45)	34.81 (1.68)	39.54 (1.40)	43.94 (1.41)	48.86 (1.96)	34.63 (3.24)

SD: standard deviation

Supplementary Table 6. Mean levels of black carbon (10^{-5}/m) in each age by latent classes.

Age	Low, decreasing (14.7%)	Average-low, decreasing (22.2%)	Average, decreasing (21.0%)	Average-high, decreasing (21.1%)	High, decreasing (13.5%)	High, decreasing fast (7.5%)
	Mean (SD) 10^{-5}/m					
0	1.34 (0.33)	1.47 (0.21)	1.75 (0.23)	2.02 (0.22)	2.22 (0.19)	2.07 (0.23)
1	1.32 (0.30)	1.48 (0.19)	1.76 (0.21)	2.04 (0.21)	2.24 (0.18)	2.07 (0.21)
2	1.15 (0.24)	1.33 (0.18)	1.61 (0.22)	1.87 (0.23)	2.06 (0.24)	1.92 (0.26)
3	1.06 (0.18)	1.26 (0.13)	1.52 (0.15)	1.78 (0.14)	1.96 (0.13)	1.73 (0.25)
4	1.21 (0.21)	1.47 (0.20)	1.77 (0.24)	2.07 (0.25)	2.28 (0.28)	1.90 (0.41)
5	1.25 (0.16)	1.54 (0.13)	1.87 (0.16)	2.19 (0.16)	2.41 (0.14)	1.93 (0.43)
6	1.04 (0.18)	1.30 (0.20)	1.59 (0.24)	1.85 (0.27)	2.05 (0.28)	1.58 (0.41)
7	0.92 (0.10)	1.15 (0.10)	1.40 (0.11)	1.62 (0.12)	1.80 (0.10)	1.29 (0.33)
8	0.97 (0.11)	1.22 (0.12)	1.48 (0.14)	1.72 (0.16)	1.90 (0.16)	1.25 (0.33)
9	1.16 (0.16)	1.47 (0.19)	1.77 (0.23)	2.05 (0.26)	2.27 (0.27)	1.40 (0.37)
10	1.15 (0.12)	1.45 (0.14)	1.77 (0.17)	2.04 (0.18)	2.29 (0.17)	1.36 (0.28)
11	1.24 (0.16)	1.57 (0.19)	1.88 (0.21)	2.18 (0.24)	2.42 (0.25)	1.38 (0.25)
12	1.10 (0.18)	1.39 (0.22)	1.69 (0.26)	1.95 (0.28)	2.19 (0.31)	1.26 (0.25)
13	1.02 (0.11)	1.29 (0.12)	1.55 (0.12)	1.78 (0.15)	2.00 (0.12)	1.13 (0.17)
14	1.06 (0.12)	1.34 (0.14)	1.61 (0.17)	1.85 (0.21)	2.08 (0.20)	1.16 (0.16)
15	1.00 (0.11)	1.26 (0.14)	1.52 (0.15)	1.74 (0.17)	1.96 (0.15)	1.11 (0.16)
16	0.92 (0.09)	1.15 (0.12)	1.39 (0.13)	1.59 (0.16)	1.80 (0.13)	1.02 (0.14)
17	0.85 (0.09)	1.07 (0.11)	1.29 (0.13)	1.47 (0.16)	1.67 (0.13)	0.95 (0.14)
18	0.94 (0.13)	1.19 (0.17)	1.42 (0.19)	1.62 (0.22)	1.82 (0.21)	1.03 (0.18)
Overall	1.09 (0.08)	1.34 (0.07)	1.61 (0.07)	1.86 (0.07)	2.07 (0.08)	1.45 (0.13)

SD: standard deviation

Supplementary Table 7. Fine particulate matter ($PM_{2.5}$) classes distribution by socioeconomic indicators.

Indicator	Low, decreasing (6.1%)	Average-low, decreasing (18.0%)	Average-high, decreasing (53.5%)	High, decreasing (21.9%)
			%	
Maternal education	p<0.001			
Low	2.6	12.9	58.8	25.7
Medium	6.0	17.5	53.5	23.0
High	11.3	22.5	50.6	15.6
Area deprivation	p<0.001			
1st Q (least deprived)	6.6	25.7	48.3	19.4
2nd quintile	11.2	22.1	51.4	15.3
3rd quintile	8.0	17.3	52.3	22.4
4th quintile	3.6	11.7	60.9	23.8
5th Q (most deprived)	2.9	11.7	56.0	29.4
Moving status from pregnancy to 18 years	p<0.001			
Moved address	26.4	37.1	30.7	5.8

$PM_{2.5}$: fine particulate matter; Q: quintile

Supplementary Table 8. Nitrogen dioxide (NO_2) classes distribution by socioeconomic indicators.

	Air pollutant classes					
	**Low, decreasing (9.7%)**	**Average-low, decreasing (17.2%)**	**Average, decreasing (26.9%)**	**Average-high, decreasing (30.0%)**	**High, decreasing (9.4%)**	**High, decreasing fast (6.8%)**
**Indicator**			**%**			
Maternal education	p<0.001					
Low	4.7	17.1	33.5	33.7	8.4	2.6
Medium	9.8	18.1	28.3	28.5	8.8	6.5
High	12.4	14.2	17.8	33.2	12.3	10.1
Area deprivation	p<0.001					
1st Q (least deprived)	11.4	25.3	28.0	26.6	3.3	5.4
2nd quintile	20.2	21.9	22.8	23.0	5.2	6.9
3rd quintile	10.5	14.2	25.9	30.4	10.7	8.3
4th quintile	3.4	11.3	24.9	39.6	14.9	5.9
5th Q (most deprived)	1.6	12.7	33.3	31.1	14.2	7.1
Moving status from pregnancy to 18 years	p<0.001					
Moved address	21.1	18.8	17.8	10.7	4.3	27.2

Q: quintile

Supplementary Table 9. Black carbon (BC) classes distribution by socioeconomic indicators.

	Air pollutant classes					
	Low, decreasing (14.7%)	Average-low, decreasing (22.2%)	Average, decreasing (21.0%)	Average-high, decreasing (21.1%)	High, decreasing (13.5%)	High, decreasing fast (7.5%)
**Indicator**			%			
Maternal education	p<0.001					
Low	7.0	27.4	26.2	21.6	14.0	3.8
Medium	14.7	23.1	22.1	20.3	12.9	6.9
High	19.2	15.9	14.2	23.5	15.4	11.8
Area deprivation	p<0.001					
1st Q (least deprived)	22.2	29.5	26.1	14.7	2.4	5.1
2nd quintile	25.0	27.1	16.5	18.0	6.5	6.9
3rd quintile	14.6	21.3	16.2	24.8	14.0	9.1
4th quintile	6.1	14.5	22.6	24.9	24.3	7.6
5th Q (most deprived)	3.5	17.9	24.8	23.0	22.1	8.7
Moving status from pregnancy to 18 years	p<0.001					
Moved address	34.3	20.8	9.7	8.6	2.6	23.9

Q: quintile

Supplementary Table 10. Association between fine particulate matter ($PM_{2.5}$) from pregnancy to 18 years with cardiovascular health indicators at age 18 years.

Outcome	Exposure	β (95% CI)	p-value
Peripheral SBP, mmHg	$PM_{2.5}$ from pregnancy-18y	0.14 (-0.30, 0.58)	0.541
	Low, decreasing	Reference	0.991
	Average-low, decreasing	0.25 (-1.39, 1.89)	
	Average-high, decreasing	0.13 (-1.37, 1.62)	
	High, decreasing	0.18 (-1.44, 1.79)	
Central SBP, mmHg	$PM_{2.5}$ from pregnancy-18y	0.31 (-0.07, 0.68)	0.108
	Low, decreasing	Reference	0.675
	Average-low, decreasing	0.52 (-0.87, 1.91)	
	Average-high, decreasing	0.53 (-0.73, 1.80)	
	High, decreasing	0.84 (-0.53, 2.21)	
Peripheral DBP, mmHg	$PM_{2.5}$ from pregnancy-18y	0.46 (0.14, 0.78)	0.005
	Low, decreasing	Reference	0.238
	Average-low, decreasing	0.61 (-0.57, 1.80)	
	Average-high, decreasing	0.69 (-0.39, 1.77)	
	High, decreasing	1.16 (-0.01, 2.33)	
Central DBP, mmHg	$PM_{2.5}$ from pregnancy-18y	0.50 (0.17, 0.83)	0.003
	Low, decreasing	Reference	0.195
	Average-low, decreasing	0.68 (-0.54, 1.89)	
	Average-high, decreasing	0.81 (-0.30, 1.91)	
	High, decreasing	1.26 (0.07, 2.46)	
CIMT, µm	$PM_{2.5}$ from pregnancy-18y	-2.28 (-4.03, -0.52)	0.011
	Low, decreasing	Reference	0.103
	Average-low, decreasing	-3.29 (-9.70, 3.11)	
	Average-high, decreasing	-4.90 (-10.69, 0.90)	
	High, decreasing	-7.39 (-13.68, -1.10)	
PWV, m/s	$PM_{2.5}$ from pregnancy-18y	0.02 (-0.01, 0.05)	0.117
	Low, decreasing	Reference	0.400
	Average-low, decreasing	0.04 (-0.06, 0.14)	
	Average-high, decreasing	0.07 (-0.02, 0.17)	
	High, decreasing	0.06 (-0.04, 0.16)	
Heart rate, bpm	$PM_{2.5}$ from pregnancy-18y	0.31 (-0.14, 0.76)	0.180
	Low, decreasing	Reference	0.468
	Average-low, decreasing	0.26 (-1.41, 1.94)	
	Average-high, decreasing	0.89 (-0.64, 2.41)	
	High, decreasing	0.44 (-1.21, 2.09)	
AIx, %	$PM_{2.5}$ from pregnancy-18y	-0.09 (-0.65, 0.48)	0.765
	Low, decreasing	Reference	0.699
	Average-low, decreasing	0.07 (-2.02, 2.16)	
	Average-high, decreasing	-0.23 (-2.14, 1.67)	
	High, decreasing	0.49 (-1.57, 2.55)	

AIx: augmentation index; CIMT: carotid media-intima thickness; DBP: diastolic blood pressure; $PM_{2.5}$: fine particulate matter; PWV: pulse wave velocity; SBP: systolic blood pressure

Supplementary Table 11. Association between nitrogen dioxide (NO_2) from pregnancy to 18 years with cardiovascular health indicators at age 18 years.

Outcome	Exposure	β (95% CI)	p-value
Peripheral SBP, mmHg	NO_2 from pregnancy-18y	0.15 (-0.35, 0.64)	0.565
	Low, decreasing	Reference	0.315
	Average-low, decreasing	-0.42 (-1.87, 1.03)	
	Average, decreasing	0.36 (-1.00, 1.73)	
	Average-high, decreasing	-0.33 (-1.67, 1.01)	
	High, decreasing	0.57 (-1.11, 2.25)	
	High, decreasing fast	1.10 (-0.71, 2.90)	
Central SBP, mmHg	NO_2 from pregnancy-18y	0.30 (-0.12, 0.72)	0.161
	Low, decreasing	Reference	0.249
	Average-low, decreasing	0.17 (-1.06, 1.41)	
	Average, decreasing	0.67 (-0.49, 1.83)	
	Average-high, decreasing	0.24 (-0.90, 1.38)	
	High, decreasing	1.21 (-0.22, 2.64)	
	High, decreasing fast	1.37 (-0.17, 2.90)	
Peripheral DBP, mmHg	NO_2 from pregnancy-18y	0.45 (0.09, 0.81)	0.015
	Low, decreasing	Reference	0.108
	Average-low, decreasing	0.85 (-0.21, 1.90)	
	Average, decreasing	0.86 (-0.14, 1.85)	
	Average-high, decreasing	0.83 (-0.15, 1.81)	
	High, decreasing	1.73 (0.50, 2.95)	
	High, decreasing fast	1.47 (0.16, 2.79)	
Central DBP, mmHg	NO_2 from pregnancy-18y	0.50 (0.13, 0.87)	0.009
	Low, decreasing	Reference	0.095
	Average-low, decreasing	0.86 (-0.22, 1.94)	
	Average, decreasing	0.94 (-0.08, 1.96)	
	Average-high, decreasing	1.00 (0.00, 1.99)	
	High, decreasing	1.78 (0.52, 3.03)	
	High, decreasing fast	1.61 (0.26, 2.95)	
CIMT, µm	NO_2 from pregnancy-18y	-0.42 (-2.41, 1.57)	0.681
	Low, decreasing	Reference	0.835
	Average-low, decreasing	-1.64 (-7.41, 4.14)	
	Average, decreasing	-1.38 (-6.85, 4.10)	
	Average-high, decreasing	-0.46 (-5.86, 4.93)	
	High, decreasing	-3.95 (-10.71, 2.80)	
	High, decreasing fast	0.07 (-7.16, 7.29)	
PWV, m/s	NO_2 from pregnancy-18y	0.01 (-0.02, 0.04)	0.659
	Low, decreasing	Reference	0.323
	Average-low, decreasing	0.08 (-0.01, 0.17)	
	Average, decreasing	0.08 (0.00, 0.17)	
	Average-high, decreasing	0.05 (-0.03, 0.14)	
	High, decreasing	0.02 (-0.09, 0.13)	
	High, decreasing fast	0.02 (-0.09, 0.14)	
Heart rate, bpm	NO_2 from pregnancy-18y	0.18 (-0.33, 0.68)	0.500
	Low, decreasing	Reference	0.195

	Average-low, decreasing	0.65 (-0.83, 2.14)	
	Average, decreasing	0.49 (-0.91, 1.89)	
	Average-high, decreasing	1.18 (-0.20, 2.55)	
	High, decreasing	-0.57 (-2.29, 1.16)	
	High, decreasing fast	0.41 (-1.44, 2.26)	
AIx, %	NO_2 from pregnancy-18y	0.02 (-0.62, 0.65)	0.956
	Low, decreasing	Reference	0.823
	Average-low, decreasing	0.60 (-1.25, 2.45)	
	Average, decreasing	0.68 (-1.07, 2.43)	
	Average-high, decreasing	-0.04 (-1.76, 1.67)	
	High, decreasing	0.84 (-1.31, 2.98)	
	High, decreasing fast	0.09 (-2.21, 2.39)	

AIx: augmentation index; CIMT: carotid media-intima thickness; DBP: diastolic blood pressure; NO_2: nitrogen dioxide; PWV: pulse wave velocity; SBP: systolic blood pressure

Supplementary Table 12. Association between black carbon (BC) from pregnancy to 18 years with cardiovascular health indicators at age 18 years.

Outcome	Exposure	β (95% CI)	p-value
Peripheral SBP, mmHg	BC from pregnancy-18y	0.10 (-0.56, 0.76)	0.772
	Low, decreasing	Reference	0.063
	Average-low, decreasing	0.26 (-0.98, 1.50)	
	Average, decreasing	0.30 (-0.95, 1.55)	
	Average-high, decreasing	1.13 (-0.14, 2.40)	
	High, decreasing	0.14 (-1.30, 1.58)	
	High, decreasing fast	2.18 (0.54, 3.83)	
Central SBP, mmHg	BC from pregnancy-18y	0.18 (-0.38, 0.74)	0.529
	Low, decreasing	Reference	0.138
	Average-low, decreasing	0.52 (-0.53, 1.58)	
	Average, decreasing	0.57 (-0.49, 1.64)	
	Average-high, decreasing	1.10 (0.02, 2.18)	
	High, decreasing	0.28 (-0.94, 1.51)	
	High, decreasing fast	1.72 (0.32, 3.12)	
Peripheral DBP, mmHg	BC from pregnancy-18y	0.32 (-0.16, 0.80)	0.195
	Low, decreasing	Reference	0.154
	Average-low, decreasing	0.89 (-0.01, 1.80)	
	Average, decreasing	0.79 (-0.12, 1.70)	
	Average-high, decreasing	1.00 (0.08, 1.93)	
	High, decreasing	0.89 (-0.16, 1.94)	
	High, decreasing fast	1.59 (0.39, 2.79)	
Central DBP, mmHg	BC from pregnancy-18y	0.41 (-0.09, 0.90)	0.106
	Low, decreasing	Reference	0.102
	Average-low, decreasing	0.95 (0.03, 1.88)	
	Average, decreasing	0.90 (-0.03, 1.83)	
	Average-high, decreasing	1.17 (0.22, 2.12)	
	High, decreasing	1.03 (-0.04, 2.11)	
	High, decreasing fast	1.70 (0.47, 2.93)	
CIMT, µm	BC from pregnancy-18y	1.06 (-1.58, 3.70)	0.431
	Low, decreasing	Reference	0.189
	Average-low, decreasing	-3.23 (-8.12, 1.65)	
	Average, decreasing	-2.33 (-7.31, 2.65)	
	Average-high, decreasing	1.99 (-3.04, 7.03)	
	High, decreasing	-0.86 (-6.64, 4.93)	
	High, decreasing fast	-4.09 (-10.66, 2.48)	
PWV, m/s	BC from pregnancy-18y	-0.02 (-0.06, 0.02)	0.336
	Low, decreasing	Reference	0.005
	Average-low, decreasing	0.14 (0.06, 0.21)	
	Average, decreasing	0.06 (-0.02, 0.14)	
	Average-high, decreasing	0.03 (-0.05, 0.11)	
	High, decreasing	0.03 (-0.06, 0.12)	
	High, decreasing fast	0.00 (-0.10, 0.11)	
Heart rate, bpm	BC from pregnancy-18y	0.68 (0.00, 1.36)	0.049
	Low, decreasing	Reference	0.159

	Average-low, decreasing	0.84 (-0.43, 2.11)	
	Average, decreasing	1.19 (-0.09, 2.47)	
	Average-high, decreasing	1.19 (-0.12, 2.49)	
	High, decreasing	1.76 (0.28, 3.24)	
	High, decreasing fast	1.99 (0.30, 3.68)	
AIx, %	BC from pregnancy-18y	-0.30 (-1.15, 0.54)	0.479
	Low, decreasing	Reference	0.546
	Average-low, decreasing	-0.60 (-2.18, 0.97)	
	Average, decreasing	-0.32 (-1.91, 1.27)	
	Average-high, decreasing	-0.25 (-1.87, 1.37)	
	High, decreasing	-1.28 (-3.11, 0.55)	
	High, decreasing fast	-1.64 (-3.73, 0.46)	

AIx: augmentation index; BC: black carbon; CIMT: carotid media-intima thickness; DBP: diastolic blood pressure; PWV: pulse wave velocity; SBP: systolic blood pressure

Supplementary Table 13. Association between air pollution at different age ranges and cardiovascular health markers at age 18 years.

Outcome	Exposure	0-5 years		6-12 years		13-18 years		p-value difference by age
		beta (95%CI)	p-value	beta (95%CI)	p-value	beta (95%CI)	p-value	
Peripheral SBP, mmHg	$PM_{2.5}$ (μg/m^3)	0.10 (-0.09, 0.28)	0.303	0.09 (-0.16, 0.34)	0.487	0.01 (-0.27, 0.29)	0.962	0.488
	NO_2 (μg/m^3)	0.01 (-0.03, 0.06)	0.546	0.02 (-0.04, 0.08)	0.585	0.00 (-0.07, 0.07)	0.996	0.644
	BC (10^{-5}/m)	0.17 (-0.94, 1.29)	0.757	0.09 (-0.99, 1.17)	0.873	0.09 (-1.07, 1.26)	0.876	0.977
Central SBP, mmHg	$PM_{2.5}$ (μg/m^3)	0.19 (0.04, 0.35)	0.015	0.20 (-0.01, 0.41)	0.066	0.10 (-0.14, 0.34)	0.418	0.190
	NO_2 (μg/m^3)	0.03 (-0.01, 0.07)	0.104	0.04 (-0.01, 0.09)	0.127	0.02 (-0.04, 0.08)	0.514	0.372
	BC (10^{-5}/m)	0.47 (-0.47, 1.42)	0.328	0.28 (-0.63, 1.20)	0.545	0.28 (-0.72, 1.27)	0.585	0.879
Peripheral DBP, mmHg	$PM_{2.5}$ (μg/m^3)	0.27 (0.13, 0.40)	<0.001	0.26 (0.08, 0.44)	0.005	0.20 (0.00, 0.41)	0.051	0.432
	NO_2 (μg/m^3)	0.05 (0.02, 0.08)	0.003	0.05 (0.01, 0.10)	0.014	0.04 (-0.01, 0.10)	0.084	0.712
	BC (10^{-5}/m)	1.01 (0.21, 1.82)	0.014	0.43 (-0.35, 1.21)	0.283	0.54 (-0.31, 1.39)	0.210	0.146
Central DBP, mmHg	$PM_{2.5}$ (μg/m^3)	0.28 (0.14, 0.42)	<0.001	0.28 (0.10, 0.47)	0.003	0.23 (0.02, 0.43)	0.033	0.455
	NO_2 (μg/m^3)	0.05 (0.02, 0.09)	0.002	0.06 (0.02, 0.10)	0.008	0.05 (0.00, 0.10)	0.065	0.669
	BC (10^{-5}/m)	1.18 (0.35, 2.01)	0.005	0.56 (-0.25, 1.36)	0.175	0.67 (-0.20, 1.54)	0.130	0.124
CIMT, μm	$PM_{2.5}$ (μg/m^3)	-1.36 (-2.09, -0.63)	<0.001	-1.16 (-2.15, -0.18)	0.021	-1.02 (-2.12, 0.09)	0.072	0.778
	NO_2 (μg/m^3)	-0.09 (-0.27, 0.09)	0.336	-0.08 (-0.31, 0.16)	0.525	-0.02 (-0.30, 0.26)	0.907	0.644
	BC (10^{-5}/m)	-0.39 (-4.8, 4.01)	0.861	1.48 (-2.83, 5.79)	0.501	1.97 (-2.7, 6.64)	0.409	0.498
PWV, m/s	$PM_{2.5}$ (μg/m^3)	0.00 (-0.01, 0.01)	0.913	0.01 (0.00, 0.03)	0.069	0.02 (0.00, 0.03)	0.097	0.090
	NO_2 (μg/m^3)	0.00 (0.00, 0.00)	0.419	0.00 (0.00, 0.01)	0.474	0.00 (0.00, 0.01)	0.210	0.102
	BC (10^{-5}/m)	-0.06 (-0.13, 0.01)	0.105	-0.03 (-0.10, 0.04)	0.427	-0.01 (-0.08, 0.07)	0.843	0.260
Heart rate, bpm	$PM_{2.5}$ (μg/m^3)	0.02 (-0.17, 0.20)	0.874	0.10 (-0.15, 0.35)	0.443	0.20 (-0.09, 0.48)	0.180	0.257
	NO_2 (μg/m^3)	0.01 (-0.03, 0.06)	0.589	0.01 (-0.05, 0.07)	0.792	0.02 (-0.05, 0.10)	0.503	0.583
	BC (10^{-5}/m)	1.14 (0.00, 2.28)	0.049	0.84 (-0.26, 1.95)	0.135	0.97 (-0.22, 2.16)	0.109	0.661
AIx, %	$PM_{2.5}$ (μg/m^3)	0.06 (-0.17, 0.30)	0.601	0.02 (-0.30, 0.34)	0.902	-0.11 (-0.46, 0.25)	0.562	0.340
	NO_2 (μg/m^3)	0.00 (-0.06, 0.06)	0.986	0.02 (-0.06, 0.09)	0.635	0.00 (-0.09, 0.09)	0.996	0.535
	BC (10^{-5}/m)	-0.44 (-1.86, 0.97)	0.538	-0.21 (-1.58, 1.17)	0.769	-0.45 (-1.93, 1.04)	0.553	0.647

AIx: augmentation index; BC: black carbon; CIMT: carotid media-intima thickness; DBP: diastolic blood pressure; NO_2: nitrogen dioxide; $PM_{2.5}$: fine particulate matter; PWV: pulse wave velocity; SBP: systolic blood pressure

Supplementary Table 14. Association between fine particulate matter ($PM_{2.5}$) and cardiovascular health markers at 18 years in males and females.

Outcome	Exposure	Males		Females		p-value sex
		beta (95%CI)	p-value	beta (95%CI)	p-value	interaction
Peripheral SBP, mmHg	$PM_{2.5}$ from pregnancy-18y	0.14 (-0.57, 0.86)	0.693	0.13 (-0.43, 0.68)	0.657	0.671
	Low, decreasing	Reference	0.638	Reference	0.867	0.871
	Average-low, decreasing	1.58 (-1.07, 4.23)		-0.77 (-2.84, 1.29)		
	Average-high, decreasing	0.77 (-1.66, 3.20)		-0.34 (-2.20, 1.53)		
	High, decreasing	0.77 (-1.84, 3.38)		-0.26 (-2.29, 1.77)		
Central SBP, mmHg	$PM_{2.5}$ from pregnancy-18y	0.28 (-0.30, 0.86)	0.342	0.32 (-0.17, 0.82)	0.204	0.799
	Low, decreasing	Reference	0.449	Reference	0.568	0.824
	Average-low, decreasing	1.47 (-0.67, 3.62)		-0.24 (-2.08, 1.59)		
	Average-high, decreasing	0.66 (-1.31, 2.62)		0.46 (-1.20, 2.12)		
	High, decreasing	1.10 (-1.02, 3.22)		0.64 (-1.17, 2.44)		
Peripheral DBP, mmHg	$PM_{2.5}$ from pregnancy-18y	0.46 (-0.04, 0.95)	0.071	0.45 (0.03, 0.87)	0.037	0.837
	Low, decreasing	Reference	0.556	Reference	0.272	0.522
	Average-low, decreasing	0.97 (-0.87, 2.81)		0.31 (-1.24, 1.87)		
	Average-high, decreasing	0.49 (-1.20, 2.18)		0.84 (-0.57, 2.24)		
	High, decreasing	1.01 (-0.81, 2.82)		1.25 (-0.28, 2.78)		
Central DBP, mmHg	$PM_{2.5}$ from pregnancy-18y	0.52 (0.01, 1.03)	0.044	0.47 (0.04, 0.90)	0.031	0.757
	Low, decreasing	Reference	0.584	Reference	0.262	0.601
	Average-low, decreasing	1.01 (-0.88, 2.90)		0.40 (-1.19, 1.99)		
	Average-high, decreasing	0.66 (-1.07, 2.39)		0.92 (-0.52, 2.35)		
	High, decreasing	1.15 (-0.71, 3.01)		1.33 (-0.23, 2.90)		
CIMT, µm	$PM_{2.5}$ from pregnancy-18y	-1.55 (-4.31, 1.20)	0.269	-2.80 (-5.07, -0.53)	0.016	0.347
	Low, decreasing	Reference	0.718	Reference	0.026	0.138
	Average-low, decreasing	3.57 (-6.5, 13.65)		-8.30 (-16.58, -0.03)		
	Average-high, decreasing	0.29 (-8.92, 9.50)		-8.44 (-15.87, -1.02)		
	High, decreasing	-0.28 (-10.25, 9.68)		-12.46 (-20.54, -4.37)		
PWV, m/s	$PM_{2.5}$ from pregnancy-18y	0.02 (-0.02, 0.07)	0.314	0.02 (-0.02, 0.05)	0.282	0.666
	Low, decreasing	Reference	0.661	Reference	0.349	0.894

	Average-low, decreasing	0.10 (-0.07, 0.27)		-0.01 (-0.14, 0.11)		
	Average-high, decreasing	0.09 (-0.06, 0.24)		0.06 (-0.06, 0.17)		
	High, decreasing	0.08 (-0.09, 0.24)		0.04 (-0.08, 0.17)		
Heart rate, bpm	$PM_{2.5}$ from pregnancy-18y	0.60 (-0.10, 1.29)	0.092	0.10 (-0.49, 0.70)	0.740	0.409
	Low, decreasing	Reference	0.055	Reference	0.945	0.267
	Average-low, decreasing	0.23 (-2.36, 2.81)		0.35 (-1.85, 2.55)		
	Average-high, decreasing	2.10 (-0.27, 4.47)		-0.03 (-2.02, 1.95)		
	High, decreasing	1.19 (-1.36, 3.74)		-0.11 (-2.27, 2.05)		
AIx, %	$PM_{2.5}$ from pregnancy-18y	-0.07 (-0.88, 0.74)	0.862	-0.11 (-0.89, 0.67)	0.776	0.896
	Low, decreasing	Reference	0.140	Reference	0.876	0.677
	Average-low, decreasing	0.41 (-2.57, 3.40)		-0.29 (-3.2, 2.63)		
	Average-high, decreasing	-1.17 (-3.91, 1.57)		0.45 (-2.19, 3.08)		
	High, decreasing	0.49 (-2.46, 3.44)		0.41 (-2.46, 3.28)		

AIx: augmentation index; CIMT: carotid media-intima thickness; DBP: diastolic blood pressure; $PM_{2.5}$: fine particulate matter; PWV: pulse wave velocity; SBP: systolic blood pressure

Supplementary Table 15. Association between nitrogen dioxide (NO_2) and cardiovascular health markers at 18 years in males and females.

Outcome	Exposure	Males beta (95%CI)	p-value	Females beta (95%CI)	p-value	p-value sex interaction
Peripheral SBP, mmHg	NO_2 from pregnancy-18y	0.64 (-0.14, 1.42)	0.108	-0.24 (-0.88, 0.40)	0.460	0.034
	Low, decreasing	Reference	0.606	Reference	0.255	0.862
	Average-low, decreasing	-0.35 (-2.61, 1.91)		-0.42 (-2.30, 1.46)		
	Average, decreasing	1.00 (-1.13, 3.14)		-0.15 (-1.92, 1.62)		
	Average-high, decreasing	0.02 (-2.09, 2.13)		-0.61 (-2.34, 1.12)		
	High, decreasing	1.25 (-1.38, 3.89)		0.04 (-2.14, 2.22)		
	High, decreasing fast	0.16 (-2.60, 2.93)		1.98 (-0.40, 4.36)		
Central SBP, mmHg	NO_2 from pregnancy-18y	0.60 (-0.03, 1.23)	0.062	0.07 (-0.50, 0.64)	0.807	0.095
	Low, decreasing	Reference	0.765	Reference	0.327	0.714
	Average-low, decreasing	0.10 (-1.73, 1.93)		0.28 (-1.40, 1.95)		
	Average, decreasing	0.93 (-0.80, 2.66)		0.46 (-1.11, 2.04)		
	Average-high, decreasing	0.55 (-1.15, 2.26)		0.00 (-1.54, 1.55)		
	High, decreasing	1.25 (-0.88, 3.38)		1.19 (-0.75, 3.13)		
	High, decreasing fast	0.81 (-1.43, 3.04)		1.86 (-0.25, 3.98)		
Peripheral DBP, mmHg	NO_2 from pregnancy-18y	0.69 (0.15, 1.23)	0.013	0.26 (-0.23, 0.74)	0.300	0.125
	Low, decreasing	Reference	0.440	Reference	0.333	0.324
	Average-low, decreasing	0.53 (-1.04, 2.10)		1.13 (-0.29, 2.55)		
	Average, decreasing	1.09 (-0.40, 2.58)		0.66 (-0.68, 2.00)		
	Average-high, decreasing	1.04 (-0.43, 2.50)		0.67 (-0.64, 1.98)		
	High, decreasing	1.78 (-0.05, 3.62)		1.65 (0.00, 3.30)		
	High, decreasing fast	1.41 (-0.52, 3.33)		1.49 (-0.31, 3.29)		
Central DBP, mmHg	NO_2 from pregnancy-18y	0.79 (0.23, 1.35)	0.006	0.26 (-0.23, 0.76)	0.296	0.088
	Low, decreasing	Reference	0.297	Reference	0.356	0.256
	Average-low, decreasing	0.56 (-1.05, 2.17)		1.13 (-0.33, 2.59)		
	Average, decreasing	1.29 (-0.23, 2.81)		0.65 (-0.72, 2.02)		
	Average-high, decreasing	1.35 (-0.16, 2.85)		0.73 (-0.61, 2.07)		
	High, decreasing	1.90 (0.02, 3.78)		1.65 (-0.04, 3.33)		

	High, decreasing fast	1.62 (-0.35, 3.59)		1.55 (-0.28, 3.39)		
CIMT, µm	NO_2 from pregnancy-18y	0.89 (-2.16, 3.94)	0.567	-1.48 (-4.11, 1.16)	0.272	0.288
	Low, decreasing	Reference	0.755	Reference	0.112	0.120
	Average-low, decreasing	1.52 (-7.20, 10.24)		-4.37 (-12.08, 3.34)		
	Average, decreasing	0.90 (-7.44, 9.24)		-3.27 (-10.53, 3.98)		
	Average-high, decreasing	-1.06 (-9.29, 7.16)		-0.22 (-7.36, 6.92)		
	High, decreasing	-0.54 (-10.80, 9.71)		-6.76 (-15.73, 2.22)		
	High, decreasing fast	-6.11 (-17.07, 4.86)		5.35 (-4.24, 14.95)		
PWV, m/s	NO_2 from pregnancy-18y	0.01 (-0.04, 0.06)	0.578	0.00 (-0.04, 0.04)	0.960	0.369
	Low, decreasing	Reference	0.757	Reference	0.405	0.265
	Average-low, decreasing	0.05 (-0.09, 0.19)		0.10 (-0.02, 0.21)		
	Average, decreasing	0.10 (-0.03, 0.23)		0.07 (-0.04, 0.18)		
	Average-high, decreasing	0.04 (-0.09, 0.18)		0.06 (-0.05, 0.16)		
	High, decreasing	0.04 (-0.13, 0.20)		0.00 (-0.14, 0.13)		
	High, decreasing fast	0.04 (-0.14, 0.22)		0.00 (-0.15, 0.15)		
Heart rate, bpm	NO_2 from pregnancy-18y	0.53 (-0.23, 1.30)	0.171	-0.13 (-0.82, 0.55)	0.702	0.232
	Low, decreasing	Reference	0.246	Reference	0.593	0.148
	Average-low, decreasing	0.54 (-1.68, 2.76)		0.74 (-1.26, 2.74)		
	Average, decreasing	0.46 (-1.63, 2.56)		0.54 (-1.34, 2.42)		
	Average-high, decreasing	1.89 (-0.17, 3.96)		0.60 (-1.24, 2.44)		
	High, decreasing	-0.23 (-2.82, 2.35)		-0.92 (-3.24, 1.40)		
	High, decreasing fast	1.12 (-1.59, 3.83)		-0.27 (-2.80, 2.25)		
AIx, %	NO_2 from pregnancy-18y	-0.42 (-1.30, 0.47)	0.358	0.38 (-0.52, 1.27)	0.407	0.321
	Low, decreasing	Reference	0.321	Reference	0.120	0.833
	Average-low, decreasing	1.80 (-0.78, 4.38)		-0.38 (-3.01, 2.25)		
	Average, decreasing	-0.45 (-2.89, 2.00)		1.54 (-0.93, 4.01)		
	Average-high, decreasing	0.44 (-1.97, 2.85)		-0.40 (-2.82, 2.01)		
	High, decreasing	-0.41 (-3.41, 2.59)		1.84 (-1.19, 4.88)		
	High, decreasing fast	1.29 (-1.87, 4.44)		-1.12 (-4.43, 2.19)		

AIx: augmentation index; CIMT: carotid media-intima thickness; DBP: diastolic blood pressure; NO_2: nitrogen dioxide; PWV: pulse wave velocity; SBP: systolic blood pressure

Supplementary Table 16. Association between black carbon (BC) and cardiovascular health markers at 18 years in males and females.

Outcome	Exposure	Males beta (95%CI)	p-value	Females beta (95%CI)	p-value	p-value sex interaction
Peripheral SBP, mmHg	BC from pregnancy-18y	0.71 (-0.33, 1.75)	0.183	-0.38 (-1.23, 0.47)	0.381	0.038
	Low, decreasing	Reference	0.334	Reference	0.076	0.239
	Average-low, decreasing	0.30 (-1.64, 2.24)		0.15 (-1.45, 1.75)		
	Average, decreasing	1.19 (-0.73, 3.11)		-0.42 (-2.06, 1.22)		
	Average-high, decreasing	1.94 (-0.03, 3.90)		0.44 (-1.22, 2.09)		
	High, decreasing	1.24 (-1.05, 3.53)		-0.71 (-2.56, 1.13)		
	High, decreasing fast	1.93 (-0.57, 4.43)		2.49 (0.30, 4.68)		
Central SBP, mmHg	BC from pregnancy-18y	0.69 (-0.15, 1.53)	0.109	-0.22 (-0.98, 0.53)	0.564	0.034
	Low, decreasing	Reference	0.482	Reference	0.166	0.259
	Average-low, decreasing	0.40 (-1.18, 1.97)		0.57 (-0.85, 2.00)		
	Average, decreasing	1.03 (-0.53, 2.59)		0.23 (-1.23, 1.69)		
	Average-high, decreasing	1.45 (-0.14, 3.05)		0.79 (-0.68, 2.27)		
	High, decreasing	1.19 (-0.67, 3.05)		-0.39 (-2.04, 1.25)		
	High, decreasing fast	1.36 (-0.67, 3.38)		2.10 (0.15, 4.05)		
Peripheral DBP, mmHg	BC from pregnancy-18y	0.78 (0.06, 1.51)	0.035	-0.07 (-0.71, 0.58)	0.840	0.030
	Low, decreasing	Reference	0.164	Reference	0.127	0.127
	Average-low, decreasing	0.72 (-0.63, 2.08)		0.97 (-0.24, 2.18)		
	Average, decreasing	1.14 (-0.20, 2.48)		0.51 (-0.73, 1.76)		
	Average-high, decreasing	1.02 (-0.35, 2.39)		0.95 (-0.30, 2.21)		
	High, decreasing	2.21 (0.61, 3.80)		-0.08 (-1.49, 1.32)		
	High, decreasing fast	1.31 (-0.43, 3.05)		1.86 (0.20, 3.52)		
Central DBP, mmHg	BC from pregnancy-18y	0.92 (0.18, 1.67)	0.015	-0.02 (-0.68, 0.64)	0.955	0.024
	Low, decreasing	Reference	0.080	Reference	0.107	0.102
	Average-low, decreasing	0.80 (-0.59, 2.18)		1.01 (-0.23, 2.25)		
	Average, decreasing	1.34 (-0.04, 2.71)		0.55 (-0.73, 1.82)		
	Average-high, decreasing	1.18 (-0.23, 2.58)		1.12 (-0.17, 2.40)		
	High, decreasing	2.51 (0.87, 4.15)		-0.06 (-1.49, 1.37)		

	High, decreasing fast	1.50 (-0.28, 3.29)		1.89 (0.19, 3.59)		
CIMT, µm	BC from pregnancy-18y	1.87 (-2.20, 5.94)	0.368	0.47 (-3.00, 3.94)	0.790	0.717
	Low, decreasing	Reference	0.751	Reference	0.208	0.944
	Average-low, decreasing	-0.72 (-8.18, 6.74)		-5.51 (-11.98, 0.95)		
	Average, decreasing	1.42 (-6.18, 9.02)		-5.44 (-12.03, 1.15)		
	Average-high, decreasing	3.66 (-4.00, 11.33)		0.45 (-6.23, 7.13)		
	High, decreasing	0.75 (-8.25, 9.75)		-2.49 (-10.05, 5.07)		
	High, decreasing fast	-3.14 (-13.22, 6.95)		-5.08 (-13.76, 3.60)		
PWV, m/s	BC from pregnancy-18y	-0.03 (-0.09, 0.04)	0.460	-0.02 (-0.07, 0.03)	0.514	0.567
	Low, decreasing	Reference	0.083	Reference	0.056	0.532
	Average-low, decreasing	0.15 (0.03, 0.27)		0.13 (0.03, 0.23)		
	Average, decreasing	0.12 (0.00, 0.25)		0.01 (-0.09, 0.11)		
	Average-high, decreasing	0.04 (-0.09, 0.16)		0.02 (-0.08, 0.12)		
	High, decreasing	0.02 (-0.13, 0.17)		0.03 (-0.09, 0.15)		
	High, decreasing fast	0.00 (-0.16, 0.17)		0.00 (-0.13, 0.13)		
Heart rate, bpm	BC from pregnancy-18y	0.87 (-0.15, 1.88)	0.095	0.49 (-0.41, 1.40)	0.286	0.675
	Low, decreasing	Reference	0.048	Reference	0.315	0.690
	Average-low, decreasing	0.69 (-1.21, 2.59)		0.90 (-0.81, 2.61)		
	Average, decreasing	1.99 (0.11, 3.87)		0.51 (-1.24, 2.26)		
	Average-high, decreasing	0.48 (-1.44, 2.40)		1.70 (-0.07, 3.47)		
	High, decreasing	3.08 (0.84, 5.32)		0.72 (-1.25, 2.69)		
	High, decreasing fast	1.70 (-0.74, 4.15)		2.17 (-0.17, 4.51)		
AIx, %	BC from pregnancy-18y	-0.66 (-1.84, 0.53)	0.278	-0.02 (-1.2, 1.17)	0.976	0.702
	Low, decreasing	Reference	0.664	Reference	0.313	0.938
	Average-low, decreasing	-0.27 (-2.47, 1.94)		-0.78 (-3.01, 1.45)		
	Average, decreasing	-1.41 (-3.59, 0.77)		0.64 (-1.65, 2.93)		
	Average-high, decreasing	-1.17 (-3.40, 1.06)		0.53 (-1.78, 2.85)		
	High, decreasing	-1.82 (-4.42, 0.78)		-0.80 (-3.37, 1.77)		
	High, decreasing fast	-1.11 (-3.96, 1.73)		-2.16 (-5.20, 0.89)		

AIx: augmentation index; BC: black carbon; CIMT: carotid media-intima thickness; DBP: diastolic blood pressure; PWV: pulse wave velocity; SBP: systolic blood pressure

Supplementary Table 17. Association between fine particulate matter ($PM_{2.5}$) and cardiovascular health markers at 18 years in non-movers and movers.

Outcome	Exposure	Non-movers		Movers		p-value
		beta (95%CI)	p-value	beta (95%CI)	p-value	interaction
Peripheral SBP, mmHg	$PM_{2.5}$ from pregnancy-18y	-0.10 (-0.66, 0.46)	0.723	0.90 (-0.07, 1.88)	0.070	0.191
	Low, decreasing	Reference	0.853	Reference	0.372	0.746
	Average-low, decreasing	-0.27 (-2.82, 2.29)		0.21 (-2.02, 2.44)		
	Average-high, decreasing	-0.69 (-3.07, 1.70)		0.89 (-1.55, 3.34)		
	High, decreasing	-0.73 (-3.20, 1.74)		3.57 (-0.61, 7.75)		
Central SBP, mmHg	$PM_{2.5}$ from pregnancy-18y	0.08 (-0.39, 0.55)	0.740	1.15 (0.30, 1.99)	0.008	0.133
	Low, decreasing	Reference	0.934	Reference	0.178	0.499
	Average-low, decreasing	0.21 (-1.96, 2.37)		0.35 (-1.59, 2.28)		
	Average-high, decreasing	-0.03 (-2.05, 2.00)		1.22 (-0.90, 3.34)		
	High, decreasing	0.21 (-1.88, 2.30)		3.80 (0.18, 7.43)		
Peripheral DBP, mmHg	$PM_{2.5}$ from pregnancy-18y	0.30 (-0.10, 0.70)	0.144	1.22 (0.47, 1.96)	0.002	0.194
	Low, decreasing	Reference	0.699	Reference	0.093	0.328
	Average-low, decreasing	0.40 (-1.43, 2.24)		0.53 (-1.17, 2.24)		
	Average-high, decreasing	0.41 (-1.30, 2.12)		0.91 (-0.96, 2.77)		
	High, decreasing	0.77 (-1.00, 2.55)		4.09 (0.90, 7.28)		
Central DBP, mmHg	$PM_{2.5}$ from pregnancy-18y	0.35 (-0.06, 0.76)	0.098	1.23 (0.46, 2.00)	0.002	0.222
	Low, decreasing	Reference	0.645	Reference	0.102	0.381
	Average-low, decreasing	0.53 (-1.35, 2.41)		0.59 (-1.17, 2.34)		
	Average-high, decreasing	0.62 (-1.13, 2.38)		0.85 (-1.07, 2.77)		
	High, decreasing	0.96 (-0.85, 2.78)		4.15 (0.87, 7.43)		
CIMT, μm	$PM_{2.5}$ from pregnancy-18y	-1.82 (-4.03, 0.38)	0.106	-5.76 (-9.77, -1.76)	0.005	0.635
	Low, decreasing	Reference	0.477	Reference	0.015	0.096
	Average-low, decreasing	2.15 (-7.79, 12.09)		-8.29 (-17.36, 0.79)		
	Average-high, decreasing	-0.20 (-9.46, 9.07)		-11.63 (-21.38, -1.87)		
	High, decreasing	-2.17 (-11.76, 7.42)		-24.75 (-41.88, -7.61)		
PWV, m/s	$PM_{2.5}$ from pregnancy-18y	0.01 (-0.03, 0.04)	0.671	0.05 (-0.01, 0.11)	0.096	0.279
	Low, decreasing	Reference	0.527	Reference	0.253	0.430

	Average-low, decreasing	0.03 (-0.13, 0.20)		0.04 (-0.09, 0.18)		
	Average-high, decreasing	0.07 (-0.08, 0.23)		0.01 (-0.13, 0.15)		
	High, decreasing	0.04 (-0.12, 0.20)		0.23 (-0.01, 0.47)		
Heart rate, bpm	$PM_{2.5}$ from pregnancy-18y	0.26 (-0.31, 0.82)	0.374	0.72 (-0.33, 1.76)	0.177	0.785
	Low, decreasing	Reference	0.250	Reference	0.443	0.808
	Average-low, decreasing	-1.15 (-3.76, 1.45)		1.87 (-0.46, 4.20)		
	Average-high, decreasing	0.05 (-2.38, 2.48)		0.79 (-1.76, 3.34)		
	High, decreasing	-0.48 (-3.00, 2.04)		1.72 (-2.65, 6.09)		
AIx, %	$PM_{2.5}$ from pregnancy-18y	-0.17 (-0.88, 0.55)	0.651	0.28 (-0.91, 1.47)	0.645	0.634
	Low, decreasing	Reference	0.408	Reference	0.590	0.694
	Average-low, decreasing	1.24 (-2.05, 4.53)		-1.25 (-3.95, 1.44)		
	Average-high, decreasing	0.37 (-2.70, 3.44)		0.54 (-2.43, 3.51)		
	High, decreasing	1.28 (-1.90, 4.46)		-0.84 (-5.86, 4.18)		

AIx: augmentation index; CIMT: carotid media-intima thickness; DBP: diastolic blood pressure; $PM_{2.5}$: fine particulate matter; PWV: pulse wave velocity; SBP: systolic blood pressure

Supplementary Table 18. Association between nitrogen dioxide (NO_2) and cardiovascular health markers at 18 years in non-movers and movers.

Outcome	Exposure	Non-movers		Movers		p-value
		beta (95%CI)	p-value	beta (95%CI)	p-value	interaction
Peripheral SBP, mmHg	NO_2 from pregnancy-18y	-0.02 (-0.59, 0.56)	0.957	0.76 (-0.45, 1.97)	0.220	0.401
	Low, decreasing	Reference	0.336	Reference	0.222	0.290
	Average-low, decreasing	-0.44 (-2.18, 1.30)		-0.75 (-3.79, 2.29)		
	Average, decreasing	0.28 (-1.36, 1.92)		-0.21 (-3.40, 2.97)		
	Average-high, decreasing	-0.34 (-1.95, 1.27)		-2.37 (-6.00, 1.27)		
	High, decreasing	0.26 (-1.67, 2.19)		4.16 (-1.24, 9.56)		
	High, decreasing fast	2.15 (-0.58, 4.87)		1.16 (-1.63, 3.96)		
Central SBP, mmHg	NO_2 from pregnancy-18y	0.13 (-0.36, 0.62)	0.591	1.01 (-0.04, 2.06)	0.058	0.295
	Low, decreasing	Reference	0.141	Reference	0.306	0.193
	Average-low, decreasing	0.18 (-1.29, 1.66)		-0.12 (-2.63, 2.39)		
	Average, decreasing	0.53 (-0.86, 1.93)		1.16 (-1.47, 3.80)		
	Average-high, decreasing	0.26 (-1.11, 1.63)		-1.49 (-4.49, 1.52)		
	High, decreasing	1.01 (-0.63, 2.65)		3.52 (-0.95, 7.98)		
	High, decreasing fast	2.88 (0.57, 5.19)		0.93 (-1.38, 3.24)		
Peripheral DBP, mmHg	NO_2 from pregnancy-18y	0.30 (-0.11, 0.72)	0.155	1.20 (0.27, 2.13)	0.012	0.224
	Low, decreasing	Reference	0.077	Reference	0.244	0.409
	Average-low, decreasing	0.73 (-0.52, 1.99)		0.99 (-1.15, 3.12)		
	Average, decreasing	0.57 (-0.61, 1.75)		2.55 (0.31, 4.79)		
	Average-high, decreasing	0.73 (-0.43, 1.89)		0.30 (-2.25, 2.85)		
	High, decreasing	1.50 (0.11, 2.89)		2.95 (-0.84, 6.75)		
	High, decreasing fast	2.64 (0.68, 4.61)		1.05 (-0.91, 3.01)		
Central DBP, mmHg	NO_2 from pregnancy-18y	0.36 (-0.07, 0.78)	0.101	1.19 (0.23, 2.15)	0.015	0.254
	Low, decreasing	Reference	0.045	Reference	0.343	0.320
	Average-low, decreasing	0.79 (-0.49, 2.08)		0.90 (-1.30, 3.10)		
	Average, decreasing	0.71 (-0.50, 1.92)		2.49 (0.18, 4.79)		
	Average-high, decreasing	0.94 (-0.25, 2.13)		0.43 (-2.20, 3.05)		
	High, decreasing	1.62 (0.20, 3.04)		2.65 (-1.25, 6.55)		

	High, decreasing fast	3.00 (1.00, 5.01)		0.98 (-1.04, 2.99)		
CIMT, µm	NO_2 from pregnancy-18y	-0.05 (-2.35, 2.24)	0.964	-2.30 (-7.31, 2.72)	0.369	0.640
	Low, decreasing	Reference	0.768	Reference	0.648	0.819
	Average-low, decreasing	-2.34 (-9.22, 4.54)		1.38 (-10.36, 13.11)		
	Average, decreasing	-1.42 (-7.94, 5.10)		-4.61 (-16.72, 7.51)		
	Average-high, decreasing	-0.32 (-6.73, 6.10)		-8.01 (-22.17, 6.15)		
	High, decreasing	-4.12 (-11.82, 3.57)		-1.49 (-21.71, 18.72)		
	High, decreasing fast	-4.17 (-15.19, 6.84)		2.59 (-8.21, 13.40)		
PWV, m/s	NO_2 from pregnancy-18y	0.00 (-0.04, 0.03)	0.846	0.02 (-0.05, 0.09)	0.613	0.449
	Low, decreasing	Reference	0.284	Reference	0.479	0.096
	Average-low, decreasing	0.09 (-0.02, 0.20)		0.10 (-0.08, 0.27)		
	Average, decreasing	0.11 (0.01, 0.21)		-0.06 (-0.24, 0.12)		
	Average-high, decreasing	0.07 (-0.03, 0.18)		-0.09 (-0.30, 0.12)		
	High, decreasing	0.04 (-0.09, 0.16)		0.02 (-0.28, 0.32)		
	High, decreasing fast	0.12 (-0.05, 0.29)		-0.04 (-0.20, 0.13)		
Heart rate, bpm	NO_2 from pregnancy-18y	0.01 (-0.58, 0.60)	0.973	0.98 (-0.31, 2.27)	0.137	0.354
	Low, decreasing	Reference	0.309	Reference	0.084	0.960
	Average-low, decreasing	0.44 (-1.34, 2.21)		0.93 (-2.12, 3.98)		
	Average, decreasing	-0.08 (-1.75, 1.60)		3.66 (0.47, 6.85)		
	Average-high, decreasing	0.84 (-0.8, 2.49)		3.00 (-0.65, 6.64)		
	High, decreasing	-0.70 (-2.67, 1.27)		-2.66 (-8.07, 2.75)		
	High, decreasing fast	0.74 (-2.03, 3.52)		0.31 (-2.49, 3.11)		
AIx, %	NO_2 from pregnancy-18y	0.08 (-0.66, 0.82)	0.834	-0.1 (-1.57, 1.36)	0.890	0.771
	Low, decreasing	Reference	0.663	Reference	0.881	0.955
	Average-low, decreasing	1.27 (-0.97, 3.51)		-0.58 (-3.98, 2.83)		
	Average, decreasing	1.40 (-0.72, 3.51)		-0.50 (-4.04, 3.04)		
	Average-high, decreasing	0.64 (-1.44, 2.72)		-0.81 (-4.86, 3.24)		
	High, decreasing	1.70 (-0.78, 4.19)		-2.77 (-8.73, 3.19)		
	High, decreasing fast	1.22 (-2.29, 4.74)		-1.75 (-4.86, 1.36)		

AIx: augmentation index; CIMT: carotid media-intima thickness; DBP: diastolic blood pressure; NO_2: nitrogen dioxide; PWV: pulse wave velocity; SBP: systolic blood pressure

Supplementary Table 19. Association between black carbon (BC) and cardiovascular health markers at 18 years in non-movers and movers.

Outcome	Exposure	Non-movers beta (95%CI)	p-value	Movers beta (95%CI)	p-value	p-value interaction
Peripheral SBP, mmHg	BC from pregnancy-18y	-0.09 (-0.84, 0.66)	0.816	0.81 (-1.11, 2.74)	0.406	0.523
	Low, decreasing	Reference	0.071	Reference	0.703	0.237
	Average-low, decreasing	0.30 (-1.20, 1.79)		-0.22 (-2.72, 2.27)		
	Average, decreasing	0.14 (-1.35, 1.63)		0.76 (-2.48, 4.00)		
	Average-high, decreasing	1.04 (-0.47, 2.55)		0.58 (-2.92, 4.08)		
	High, decreasing	-0.07 (-1.73, 1.60)		1.55 (-4.40, 7.50)		
	High, decreasing fast	2.83 (0.57, 5.09)		1.81 (-0.58, 4.21)		
Central SBP, mmHg	BC from pregnancy-18y	-0.01 (-0.64, 0.62)	0.974	1.00 (-0.67, 2.67)	0.241	0.407
	Low, decreasing	Reference	0.122	Reference	0.886	0.264
	Average-low, decreasing	0.50 (-0.77, 1.77)		0.48 (-1.69, 2.66)		
	Average, decreasing	0.51 (-0.76, 1.77)		0.54 (-2.29, 3.36)		
	Average-high, decreasing	0.99 (-0.29, 2.27)		1.37 (-1.68, 4.42)		
	High, decreasing	0.13 (-1.29, 1.54)		1.11 (-4.07, 6.30)		
	High, decreasing fast	2.42 (0.50, 4.34)		1.24 (-0.85, 3.33)		
Peripheral DBP, mmHg	BC from pregnancy-18y	0.19 (-0.35, 0.73)	0.482	0.98 (-0.50, 2.46)	0.195	0.430
	Low, decreasing	Reference	0.512	Reference	0.335	0.704
	Average-low, decreasing	0.62 (-0.46, 1.70)		1.84 (-0.09, 3.77)		
	Average, decreasing	0.69 (-0.39, 1.76)		0.29 (-2.21, 2.79)		
	Average-high, decreasing	0.80 (-0.29, 1.89)		1.28 (-1.42, 3.98)		
	High, decreasing	0.65 (-0.55, 1.86)		1.99 (-2.60, 6.58)		
	High, decreasing fast	1.64 (0.01, 3.27)		1.77 (-0.07, 3.62)		
Central DBP, mmHg	BC from pregnancy-18y	0.29 (-0.26, 0.84)	0.304	0.99 (-0.53, 2.52)	0.200	0.468
	Low, decreasing	Reference	0.346	Reference	0.365	0.606
	Average-low, decreasing	0.72 (-0.39, 1.82)		1.85 (-0.14, 3.83)		
	Average, decreasing	0.85 (-0.25, 1.95)		0.14 (-2.43, 2.71)		
	Average-high, decreasing	1.00 (-0.11, 2.12)		1.44 (-1.33, 4.22)		
	High, decreasing	0.84 (-0.39, 2.08)		1.90 (-2.82, 6.62)		

	High, decreasing fast	1.88 (0.21, 3.55)		1.71 (-0.19, 3.61)		
CIMT, µm	BC from pregnancy-18y	1.72 (-1.24, 4.68)	0.255	-2.48 (-10.36, 5.41)	0.537	0.465
	Low, decreasing	Reference	0.229	Reference	0.663	0.917
	Average-low, decreasing	-2.59 (-8.44, 3.26)		-5.39 (-15.55, 4.77)		
	Average, decreasing	-2.32 (-8.18, 3.55)		3.53 (-10.09, 17.15)		
	Average-high, decreasing	2.85 (-3.07, 8.78)		-3.06 (-17.34, 11.22)		
	High, decreasing	-0.22 (-6.84, 6.41)		0.73 (-22.64, 24.10)		
	High, decreasing fast	-2.88 (-11.94, 6.18)		-6.45 (-16.30, 3.41)		
PWV, m/s	BC from pregnancy-18y	-0.04 (-0.09, 0.01)	0.111	-0.01 (-0.13, 0.10)	0.851	0.402
	Low, decreasing	Reference	0.016	Reference	0.229	0.051
	Average-low, decreasing	0.15 (0.05, 0.24)		0.11 (-0.03, 0.26)		
	Average, decreasing	0.07 (-0.02, 0.17)		-0.02 (-0.22, 0.17)		
	Average-high, decreasing	0.04 (-0.05, 0.14)		-0.09 (-0.30, 0.12)		
	High, decreasing	0.03 (-0.08, 0.13)		0.12 (-0.23, 0.47)		
	High, decreasing fast	0.08 (-0.06, 0.22)		-0.07 (-0.22, 0.07)		
Heart rate, bpm	BC from pregnancy-18y	0.66 (-0.10, 1.42)	0.087	1.15 (-0.90, 3.19)	0.270	0.891
	Low, decreasing	Reference	0.363	Reference	0.541	0.973
	Average-low, decreasing	0.72 (-0.80, 2.24)		1.42 (-1.24, 4.08)		
	Average, decreasing	1.21 (-0.31, 2.73)		0.32 (-3.13, 3.77)		
	Average-high, decreasing	1.08 (-0.46, 2.62)		2.57 (-1.15, 6.29)		
	High, decreasing	1.76 (0.06, 3.46)		-0.40 (-6.73, 5.93)		
	High, decreasing fast	1.94 (-0.37, 4.25)		2.09 (-0.46, 4.64)		
AIx, %	BC from pregnancy-18y	-0.26 (-1.22, 0.70)	0.600	-0.56 (-2.88, 1.75)	0.632	0.849
	Low, decreasing	Reference	0.965	Reference	0.063	0.966
	Average-low, decreasing	-0.28 (-2.20, 1.65)		-1.10 (-4.11, 1.91)		
	Average, decreasing	0.02 (-1.90, 1.94)		-0.33 (-4.19, 3.52)		
	Average-high, decreasing	-0.11 (-2.05, 1.83)		2.47 (-1.74, 6.68)		
	High, decreasing	-0.76 (-2.90, 1.38)		-6.59 (-13.66, 0.49)		
	High, decreasing fast	-0.55 (-3.46, 2.36)		-3.03 (-5.89, -0.16)		

AIx: augmentation index; BC: black carbon; CIMT: carotid media-intima thickness; DBP: diastolic blood pressure; PWV: pulse wave velocity; SBP: systolic blood pressure

Supplementary Table 20. Association between air pollutants from pregnancy to 18 years with birthweight and maternal pre-pregnancy body mass index (BMI).

Exposure		Birthweight		Maternal BMI	
		β (95% CI)	p-value	β (95% CI)	p-value
$PM_{2.5}$	$PM_{2.5}$ from pregnancy-18y	-0.01 (-0.03, 0.01)	0.577	0.06 (-0.08, 0.21)	0.400
	Low, decreasing	Reference	0.470	Reference	0.607
	Average-low, decreasing	-0.06 (-0.13, 0.02)		0.16 (-0.37, 0.70)	
	Average-high, decreasing	-0.04 (-0.11, 0.03)		0.28 (-0.20, 0.77)	
	High, decreasing	-0.05 (-0.12, 0.03)		0.15 (-0.37, 0.68)	
NO_2	NO_2 from pregnancy-18y	-0.01 (-0.03, 0.01)	0.391	-0.01 (-0.17, 0.16)	0.943
	Low, decreasing	Reference	0.752	Reference	0.240
	Average-low, decreasing	0.02 (-0.05, 0.08)		0.20 (-0.29, 0.68)	
	Average, decreasing	-0.02 (-0.08, 0.05)		-0.06 (-0.52, 0.39)	
	Average-high, decreasing	0.00 (-0.06, 0.06)		0.03 (-0.41, 0.48)	
	High, decreasing	-0.03 (-0.11, 0.05)		-0.13 (-0.69, 0.43)	
	High, decreasing fast	-0.02 (-0.10, 0.07)		-0.50 (-1.10, 0.10)	
BC	BC from pregnancy-18y	-0.01 (-0.04, 0.02)	0.678	0.04 (-0.18, 0.26)	0.696
	Low, decreasing	Reference	0.714	Reference	0.148
	Average-low, decreasing	0.02 (-0.04, 0.07)		0.17 (-0.24, 0.57)	
	Average, decreasing	-0.02 (-0.08, 0.03)		-0.12 (-0.54, 0.30)	
	Average-high, decreasing	0.00 (-0.06, 0.05)		-0.15 (-0.57, 0.27)	
	High, decreasing	0.00 (-0.07, 0.07)		0.24 (-0.24, 0.72)	
	High, decreasing fast	0.02 (-0.05, 0.10)		-0.36 (-0.91, 0.18)	

BC: black carbon; BMI: body mass index; NO_2: nitrogen dioxide; $PM_{2.5}$: fine particulate matter

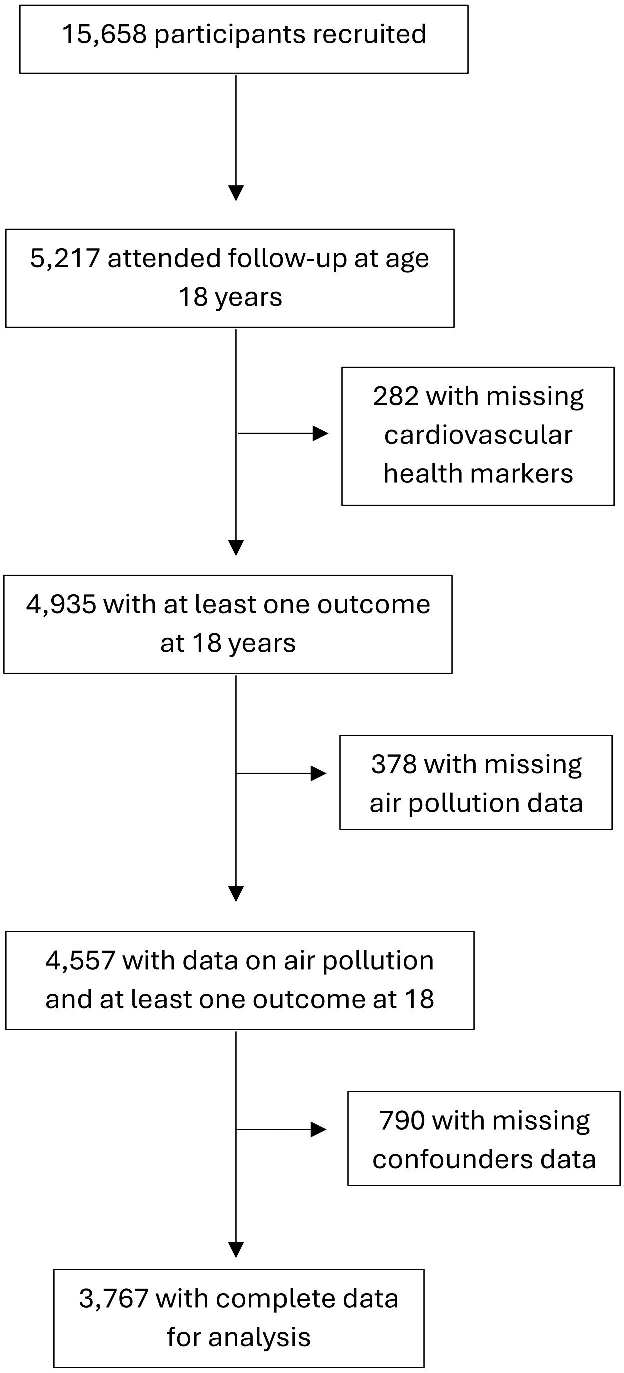

Supplementary Figure 1. Flow chart of participants.

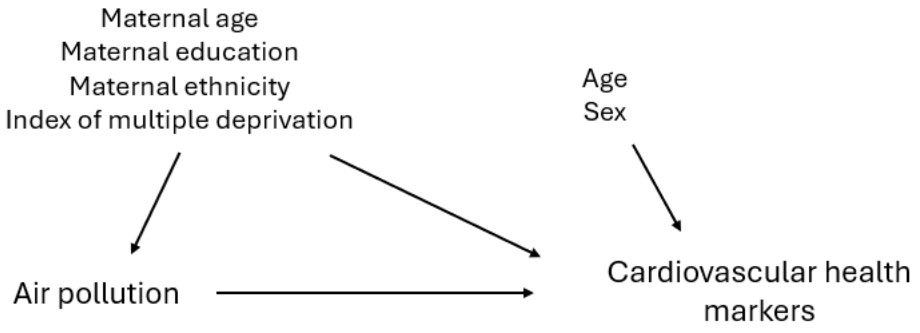

Supplementary Figure 2. Assumed causal diagram of the association between air pollution and cardiovascular health markers

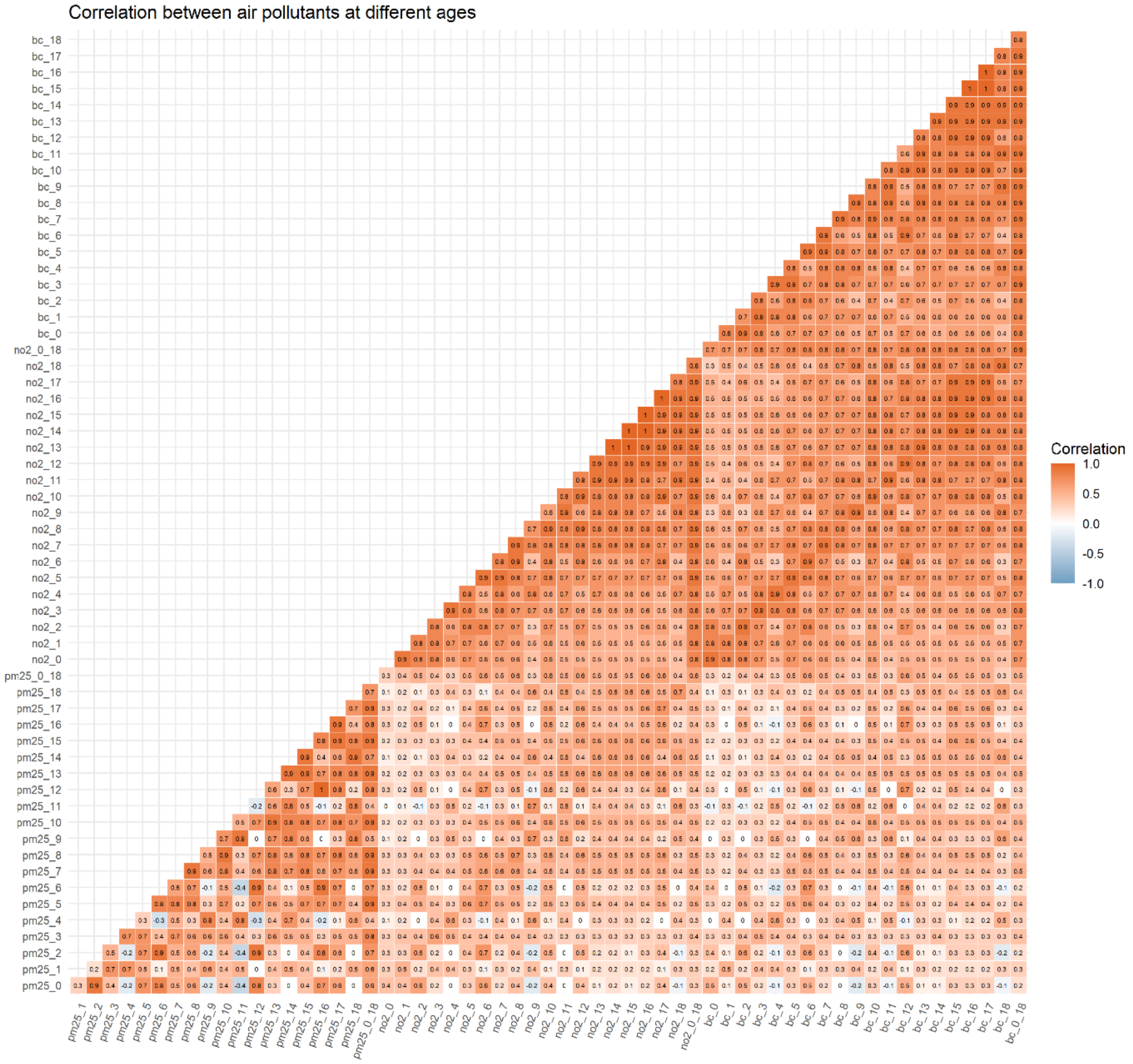

Supplementary Figure 3. Correlation between air pollutants across ages from pregnancy (age 0) to 18 years.

bc: black carbon; no2: nitrogen dioxide; pm25: fine particulate matter

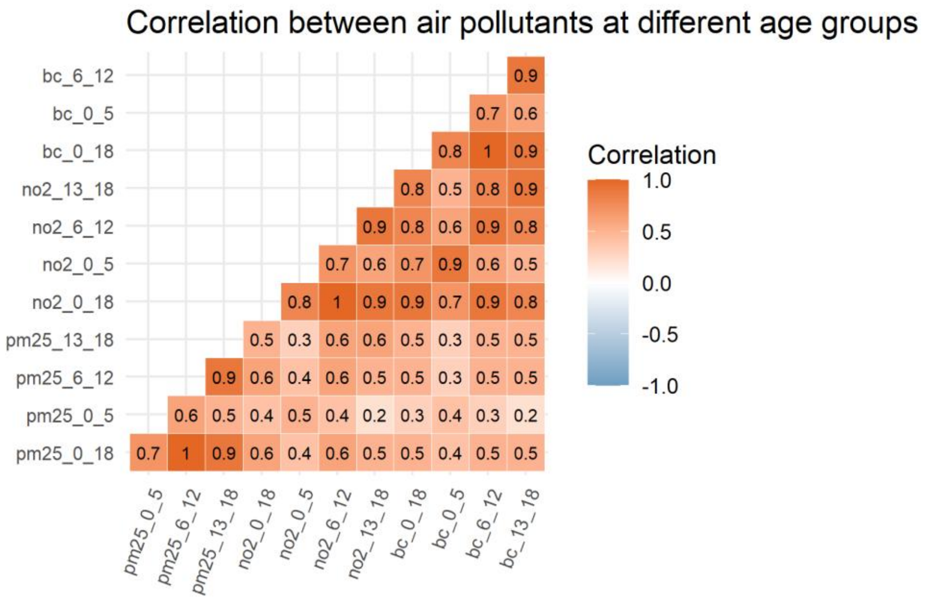

Supplementary Figure 4. Correlation between air pollutants across age ranges.

bc: black carbon; no2: nitrogen dioxide; pm25: fine particulate matter

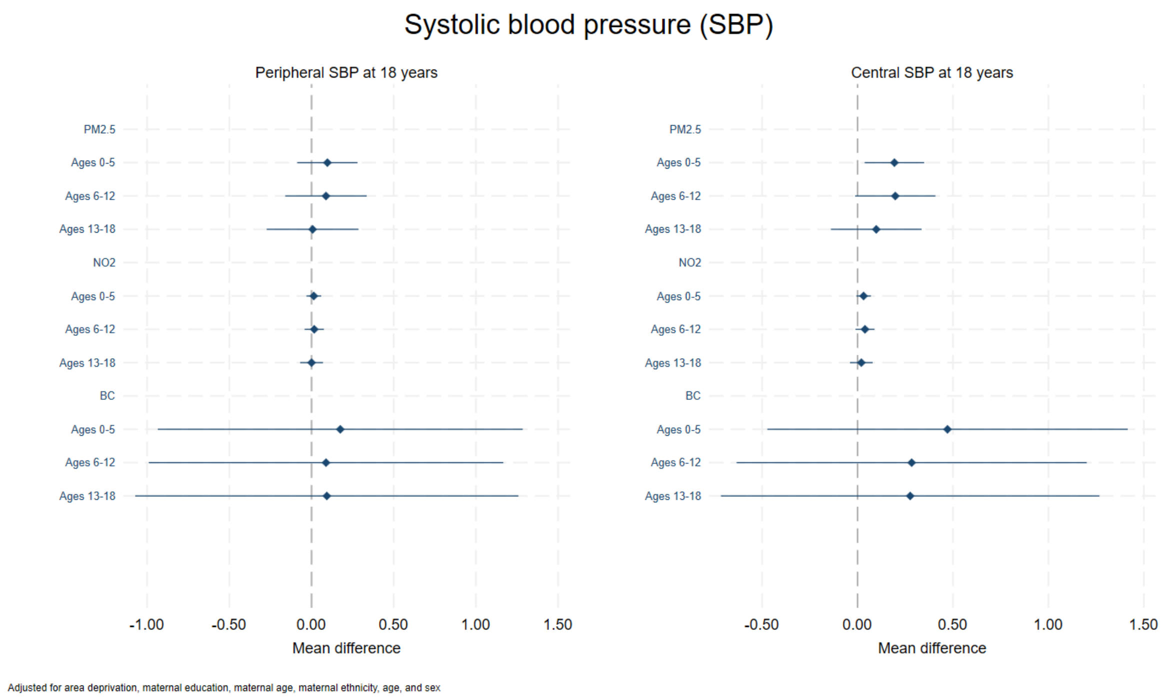
Systolic blood pressure (SBP)
Peripheral SBP at 18 years
Central SBP at 18 years
PM2.5
Ages 0-5
Ages 6-12
Ages 13-18
NO2
Ages 0-5
Ages 6-12
Ages 13-18
BC
Ages 0-5
Ages 6-12
Ages 13-18
-1.00
-0.50
0.00
0.50
1.00
1.50
Mean difference
-0.50
0.00
0.50
1.00
1.50
Mean difference
Adjusted for area deprivation, maternal education, maternal age, maternal ethnicity, age, and sex

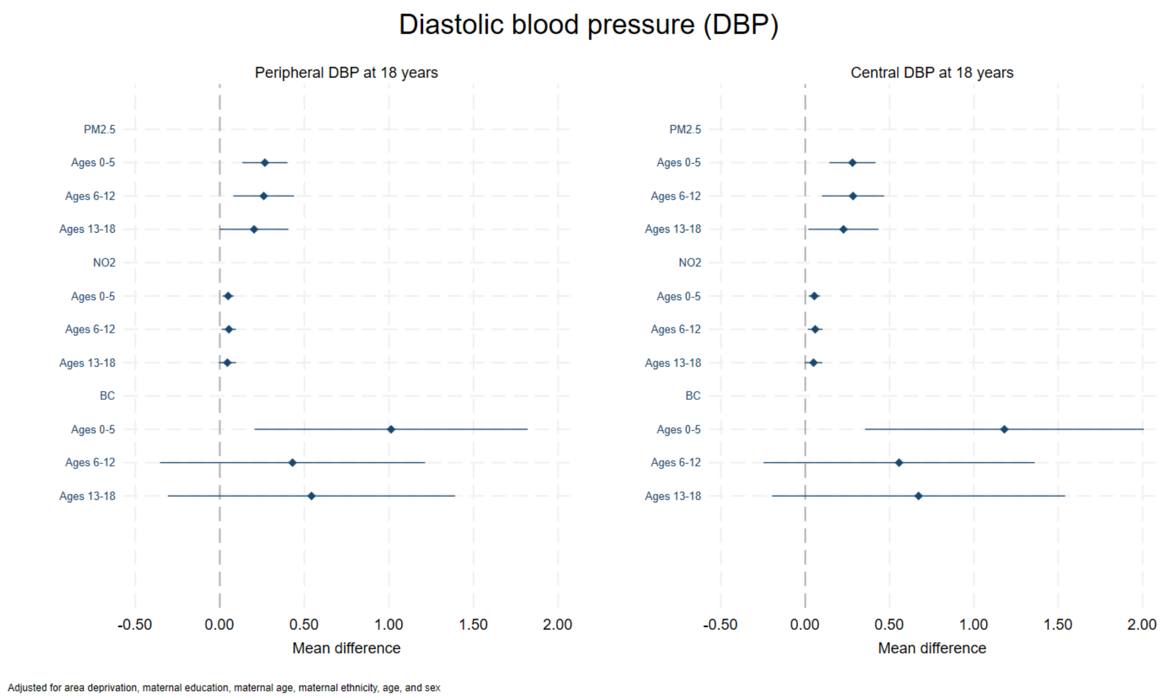
Diastolic blood pressure (DBP)
Peripheral DBP at 18 years
Central DBP at 18 years
PM2.5
Ages 0-5
Ages 6-12
Ages 13-18
NO2
Ages 0-5
Ages 6-12
Ages 13-18
BC
Ages 0-5
Ages 6-12
Ages 13-18
-0.50
0.00
0.50
1.00
1.50
2.00
Mean difference
-0.50
0.00
0.50
1.00
1.50
2.00
Mean difference
Adjusted for area deprivation, maternal education, maternal age, maternal ethnicity, age, and sex

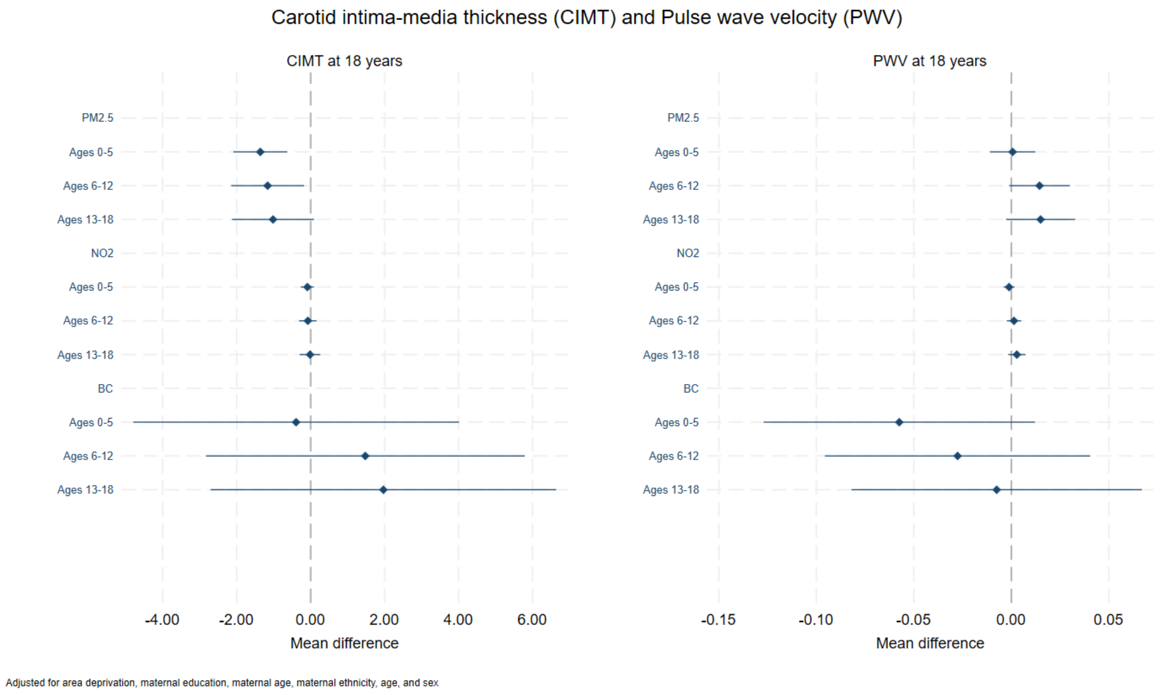

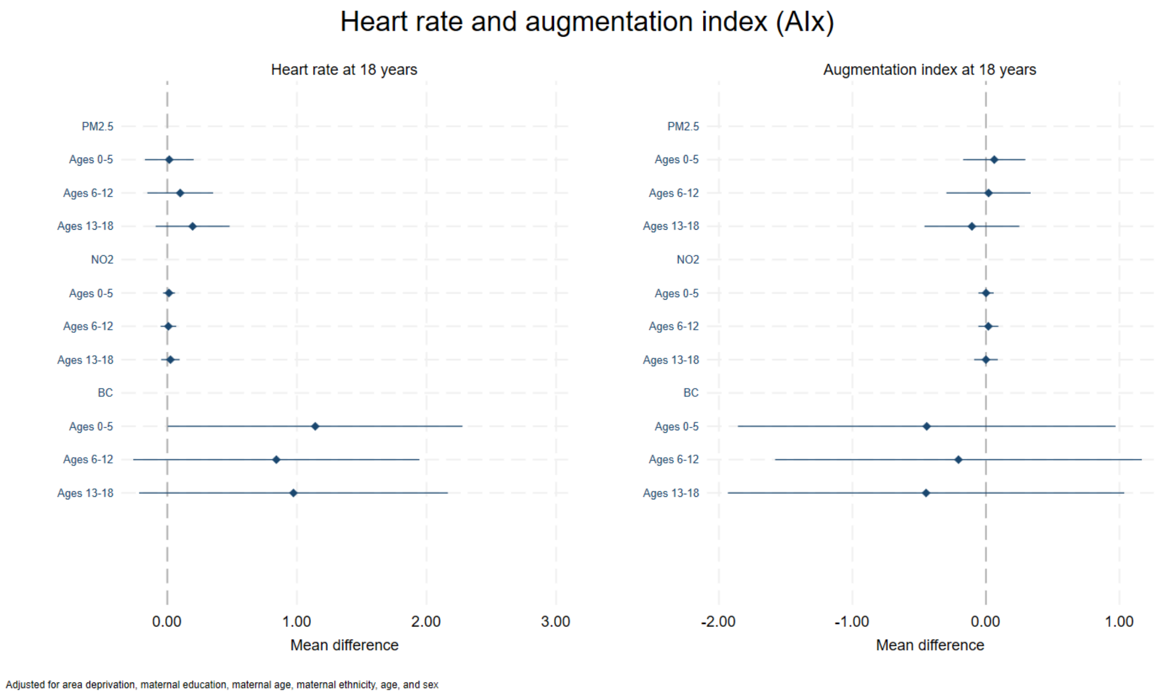

Supplementary Figure 5. Association between air pollution at different age ranges and cardiovascular health markers at age 18 years.

Estimates are mean difference in the outcome per unit increase in the exposure in each age period.
BC: black carbon; BMI: body mass index; NO_2: nitrogen dioxide; $PM_{2.5}$: fine particulate matter
